# Mediator complex proximal Tail subunit MED30 is critical for Mediator core stability and cardiomyocyte transcriptional network

**DOI:** 10.1371/journal.pgen.1009785

**Published:** 2021-09-10

**Authors:** Changming Tan, Siting Zhu, Zee Chen, Canzhao Liu, Yang E. Li, Mason Zhu, Zhiyuan Zhang, Zhiwei Zhang, Lunfeng Zhang, Yusu Gu, Zhengyu Liang, Thomas G. Boyer, Kunfu Ouyang, Sylvia M. Evans, Xi Fang

**Affiliations:** 1 Department of Medicine, University of California, San Diego, California, United States of America; 2 Department of Cardiovascular Surgery, the Second Xiangya Hospital, Central South University, Changsha, Hunan, China; 3 Department of Cardiovascular Surgery, Peking University Shenzhen Hospital, School of Chemical Biology and Biotechnology, State Key Laboratory of Chemical Oncogenomics, Peking University Shenzhen Graduate School, Shenzhen, China; 4 Department of Molecular Medicine, University of Texas Health Science Center, San Antonio, Texas, United States of America; 5 Department of Pharmacology, University of California, San Diego, California, United States of America; 6 Skaggs School of Pharmacy and Pharmaceutical Sciences, University of California, San Diego, California, United States of America; Indiana University Purdue University at Indianapolis, UNITED STATES

## Abstract

Dysregulation of cardiac transcription programs has been identified in patients and families with heart failure, as well as those with morphological and functional forms of congenital heart defects. Mediator is a multi-subunit complex that plays a central role in transcription initiation by integrating regulatory signals from gene-specific transcriptional activators to RNA polymerase II (Pol II). Recently, Mediator subunit 30 (MED30), a metazoan specific Mediator subunit, has been associated with Langer-Giedion syndrome (LGS) Type II and Cornelia de Lange syndrome-4 (CDLS4), characterized by several abnormalities including congenital heart defects. A point mutation in MED30 has been identified in mouse and is associated with mitochondrial cardiomyopathy. Very recent structural analyses of Mediator revealed that MED30 localizes to the proximal Tail, anchoring Head and Tail modules, thus potentially influencing stability of the Mediator core. However, *in vivo* cellular and physiological roles of MED30 in maintaining Mediator core integrity remain to be tested. Here, we report that deletion of MED30 in embryonic or adult cardiomyocytes caused rapid development of cardiac defects and lethality. Importantly, cardiomyocyte specific ablation of MED30 destabilized Mediator core subunits, while the kinase module was preserved, demonstrating an essential role of MED30 in stability of the overall Mediator complex. RNAseq analyses of constitutive cardiomyocyte specific *Med30* knockout (cKO) embryonic hearts and inducible cardiomyocyte specific *Med30* knockout (icKO) adult cardiomyocytes further revealed critical transcription networks in cardiomyocytes controlled by Mediator. Taken together, our results demonstrated that MED30 is essential for Mediator stability and transcriptional networks in both developing and adult cardiomyocytes. Our results affirm the key role of proximal Tail modular subunits in maintaining core Mediator stability *in vivo*.

## Author summary

MED30 has been associated with congenital heart defects. A point mutation in MED30 has been identified in mouse and is associated with mitochondrial cardiomyopathy. However, the specific role of MED30 in cardiomyocytes remains largely unknown. We found that loss of MED30 resulted in destabilization of the MED core. Our constitutive (cKO) and inducible (icKO) cardiomyocyte specific *Med30* knockout mouse models provided us with a unique opportunity to study the role of the intact MED complex in developing heart and adult heart, respectively. We further demonstrated the essential role of MED30 in maintaining specific components of the cardiac gene regulatory network and normal cardiac development and function.

## Introduction

Cardiac morphogenesis and maintenance of cardiac physiology require complex and well-orchestrated transcription programs [1–3]. Dysregulation of cardiac transcription has been identified in patients and families with heart disease [4–7]. Mediator is an evolutionarily conserved protein complex that plays a central role in transcription initiation by integrating regulatory signals from gene-specific transcriptional activators to RNA polymerase II (Pol II) [8–12]. Mediator is composed of approximately 30 subunits arranged into structurally distinct modules, including the core and kinase (CDK) modules [13–15]. The Mediator core contains 26 subunits and consists of three sub-modules: the Head, Middle, and Tail. The ‘Head’ and ‘Middle’ are essential for viability and Mediator interaction with the Pol II machinery, whereas the ‘Tail’ interacts with various activators at enhancer regions [16,17]. In the current “molecular bridge” model, Mediator serves as a central scaffold within the pre-initiation complex (PIC) to integrate regulatory signals from DNA-binding transcription factors (TFs) directly to RNA Pol II. Structural studies of metazoan Mediator have long been hampered by its large size and flexibility, and have therefore been focused on the yeast complex, easier to purify in amounts required for structural characterization [18]. Previous structural analysis of yeast Mediator has provided an understanding of the conserved core of the complex and its interaction with Pol II but failed to reveal the structure of the Tail module that contains the majority of subunits that interact with transcriptional factors([18]. Moreover, the location of metazoan-specific subunits was unknown. Cryo-electron microscopy (Cryo-EM) has enabled unprecedented progress in the quest to reveal the structure of the whole Mediator within the transcription preinitiation complex [18]. Five recent studies [19–23] have now visualized the whole Mediator to near-atomic resolution. A groundbreaking discovery from recent Cryo-EM studies was the revelation of the previously unresolved Tail module, which includes several metazoan specific subunits [19–23]. In particular, subunits MED27, MED29 and metazoan specific subunits MED28 and MED30, were observed to form the proximal part of the Tail module (‘Proximal Tail’, also called “Upper Tail”), anchoring on the Head module and displaying a large interface with the core Mediator. The newly revealed structure suggests that the subunits of the proximal Tail potentially influence the stability and conformation of the core Mediator [19–23]. These important new insights into Mediator structure are suggestive of cellular and physiological roles of the Mediator subunits in the proximal Tail, that remain to be functionally tested.

Genetic studies have identified mutations in Mediator subunits associated with cardiac disease in humans [24–33]. However, the specific role of the Mediator in cardiac development and function remains largely unknown. Recently, Mediator subunit 30 (MED30, also named TRAP25) has been associated with Langer-Giedion syndrome (LGS) Type II and Cornelia de Lange syndrome-4 (CDLS4), characterized by multiple congenital anomalies including heart defects [29,34]. Specifically, patients with LGS and CDLS4 have a 2.88-Mb deletion of 8q23.3eq24.13 encompassing MED30 [29,34]. MED30 has recently been assigned to the proximal Tail and modeled de novo [19–23,35]. The C-terminal α-helices of MED30, together with the C-terminus of MED28, form a helical bundle with the extended C-terminal helices in Head subunits MED11 and MED22, forming a convoluted interface between Tail and Head [19–23,35]. However, it remains unknown whether MED30 plays an important role in maintaining the integrity of the whole Mediator complex, or a specific submodule, or in maintaining some specific interactions between particular subunits. A dilated cardiomyopathy (DCM) phenotype in a recessive N-ethyl-N-nitrosurea (ENU)-induced mouse mutant (*zeitgeist (zg)*) has been attributed to a missense mutation in *Med30* [36]. The DCM phenotype was mapped to a single nonsynonymous A-to-T transversion in the first exon of *Med30* (herein referred to as *Med30*^zg^ allele), which resulted in an isoleucine to phenylalanine substitution at amino acid 44 of the 178-residue protein. Complementation studies between a *Med30* null allele and the *Med30*^zg^ allele did not produce any compound heterozygotes at birth, suggesting that the *zeitgeist* mutation is hypomorphic and that global deletion of *Med30* caused embryonic lethality. The hypomorphic *Med30*^zg/zg^ mutants develop progressive DCM after four weeks of age, and display premature lethality by seven weeks of age, while no evident abnormalities are observed in other organs [36]. While the overall Mediator complex was maintained intact in *Med30*^zg/zg^ mutant hearts, a specific decrease in the expression of mitochondrial genes in *Med30*^zg/zg^ mutants was postulated to account for the observed DCM, as further supported by amelioration of the DCM phenotype by feeding mice a ketogenic diet [36]. Given that the structural analysis suggests a critical role of MED30 in Mediator structure, it is perhaps puzzling that the mutation did not disrupt Mediator integrity, and only affected mitochondrial genes in mouse heart. Thus, to unequivocally study the cellular and physiological role of MED30 *in vivo*, a true *Med30* knockout mouse model is essential.

In this study, we have generated an *Med30* floxed mouse model and specifically deleted MED30 in developing or adult cardiomyocytes. Our results reveal that loss of MED30 caused degradation of multiple subunits in the Mediator core, while the kinase module was preserved, demonstrating an essential role of MED30 in the stability of the overall Mediator complex, and in cardiomyocyte development and function. We also observed that deletion of MED30 in developing or adult cardiomyocytes results in rapid development of cardiac defects and lethality. Furthermore, we utilized MED30 cardiac specific knockout mice to investigate the Mediator-regulated transcriptome in both developing and adult cardiomyocytes. Our results are consistent with the role suggested by recent structural studies for Mediator Tail subunits in mediating integrity of the Mediator core.

## Results

### Loss of MED30 in developing cardiomyocytes results in early embryonic lethality

**Fig 1:**
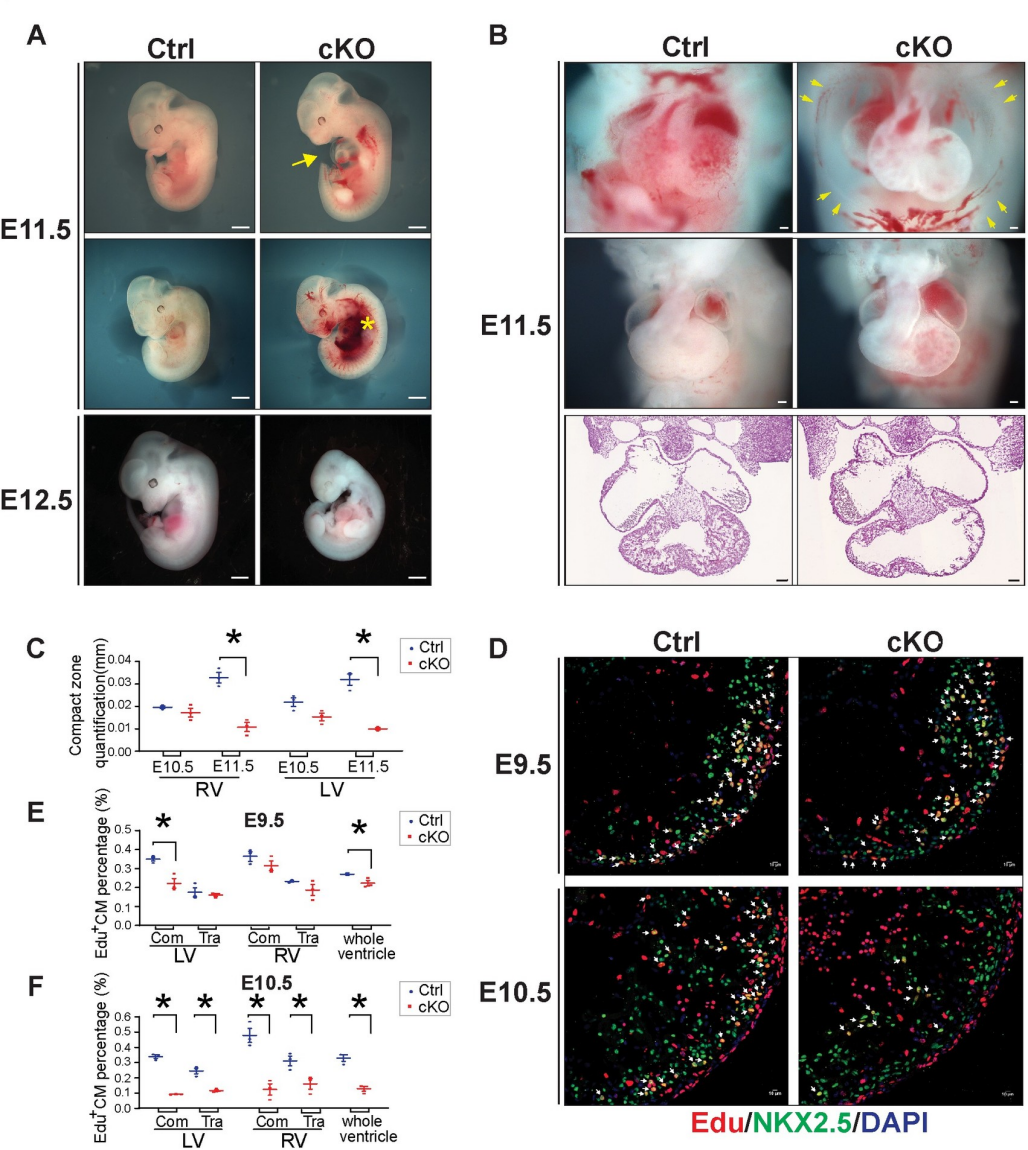
MED30 is essential for early cardiac development. **(A)** Whole embryonic morphology of constitutive cardiomyocyte-specific knockout (cKO) and control (Ctrl) littermates at E11.5 and E12.5. Arrow: edema chest; Asterisk: hemorrhage. Scale bar: 1 mm. n = 3. **(B)** Cardiac morphology (Top and middle) and H&E images (Bottom) of *Med30* cKO embryos and Ctrl littermates at E11.5. Arrow: edema chest. Scale bar: 100 μm. n = 3. **(C)** Quantitation of compact zone thickness in *Med30* cKO and Ctrl hearts at E10.5 and E11.5. RV: right ventricle; LV: left ventricle. n = 3. **(D)** Representative immunostaining images of EdU-labeled (red) heart sections from Med30 cKO and Ctrl mice at E9.5 and E10.5, using an antibody against NKX2.5 as cardiomyocyte marker (green). DNA is stained with DAPI (blue). Arrow: Edu-positive cardiomyocytes. Scale bar: 10 μM. **(E-F)** Quantification (%) of Edu-positive cardiomyocytes (CM) in (E) E9.5 and (F) E10.5 Ctrl (blue) vs. *Med30* cKO (red) heart areas as indicated. Com: compact zone; Tra: trabeculae. n = 3. Data are represented as the mean ± SEM. *P < 0.05, by 2-tailed Student’s t test.

To unequivocally study the specific role of MED30 in cardiomyocytes, we generated a floxed *Med30* mouse line with the first exon of *Med30* flanked with two loxP sites (S1A–S1C Fig). Homozygous floxed *Med30* mice (*Med30*^*f/f*^) were viable and born at expected Mendelian ratios with no observed defects. We next utilized Troponin T-Cre (*Tnnt*-Cre) [37] to ablate *Med30* in early developing cardiomyocytes. Western blot analysis confirmed the absence of MED30 in embryonic hearts isolated from *Tnnt*-Cre^+^; *Med30*^*f/f*^ mutants (hereafter referred to as *Med30* cKOs) at embryonic day (E) 9.5, compared to Cre negative control littermates (Ctrl) (S1D–S1E Fig). *Med30* cKO mutants were never recovered after birth (Table 1), indicating prenatal lethality of mutant embryos. Notably, *Med30* cKO embryos were recovered at expected Mendelian frequencies until E11.5 (Table 1). However, at E12.5, all *Med30* cKO embryos were found dead or partially resorbed (Table 1 and Fig 1A), while only 2 out of 30 cKO mutants were found dead at E11.5.

**Table 1:** Genotypic analysis of embryos from Cre^+^*Med30*^*f/+*^ male cross with Cre^*-*^*Med30*^*f/f*^ female.

Day of analysis	No. of genotype				Total
	*Cre* ^*-*^ *Med30* ^*f/+*^	*Cre* ^*-*^ *Med30* ^*f/f*^	*Cre* ^*+*^ *Med30* ^*f/+*^	*Cre* ^*+*^ *Med30* ^*f/f*^	
**E10.5**	40(30.5%)	31(23.7%)	29(22.1%)	31(23.7%)	131
**E11.5**	22(21.2%)	28(26.9%)	24(23.1%)	30(28.8%) ^a^^,^^b^^,^^c^^,^^d^^,^^e^	104
**E12.5**	22 (30.1%)	20(27.4%)	17(23.3%)	14(19.2%)^f^	73
**P1** ^**g**^	14(24.6%)	19(33.3%)	24(42.1%)	0 (0.0%)	57

^a^ 20 of these embryos have thinner ventricular walls

^b^ 4 of these embryos have smaller hearts

^c^ 3 of these embryos have chest edema

^d^ 7 of these embryos are hemorrhage

^e^ 2 of these embryos are dead

^f^ These embryos are all dead or partly absorbed

^g^ P1, postnatal day 1.

At E11.5, severe cardiac development defects were observed in *Med30* cKO hearts, including overall smaller hearts, thinner ventricular walls, chest edema, and hemorrhage (Fig 1A and 1B), while at E10.5, cKO mutants were grossly indistinguishable from control (Ctrl) littermates (S2A Fig). Hematoxylin/eosin staining revealed dramatically decreased thickness of the compact zone in both the right ventricle (RV) and left ventricle (LV) of cKO mutants at E11.5, but not E10.5, compared to control embryos (Figs 1B–1C and S2A). To investigate whether a decrease in cardiomyocyte proliferation or increased apoptosis could account for smaller hearts and thinner ventricular walls in mutant embryos, we performed EdU pulse labelling and TUNEL staining to examine cardiomyocyte proliferation and apoptosis in *Med30* cKO and control hearts. Our results revealed that the percentage of EdU positive cardiomyocytes started to decrease in compact zone of the LV of cKO mutants at E9.5, while the trabecular cardiomyocytes and RV cardiomyocytes showed no differences in EdU incorporation between control and cKOs (Fig 1D and 1E). At E10.5, cardiomyocyte proliferation was dramatically decreased in the compact zone and trabeculae of both ventricles in *Med30 cKO* hearts relative to controls (Fig 1D and 1F), suggesting a critical role for MED30 in cardiomyocyte proliferation. However, we did not observe apoptosis, as indicated by TUNEL positive cardiomyocytes, in either *Med30* cKO or control hearts at E11.5 (S2B Fig). Taken together, these results demonstrate that MED30 is essential for cardiomyocyte proliferation and cardiac development.

### MED30 is essential for Mediator stability

**Fig 2:**
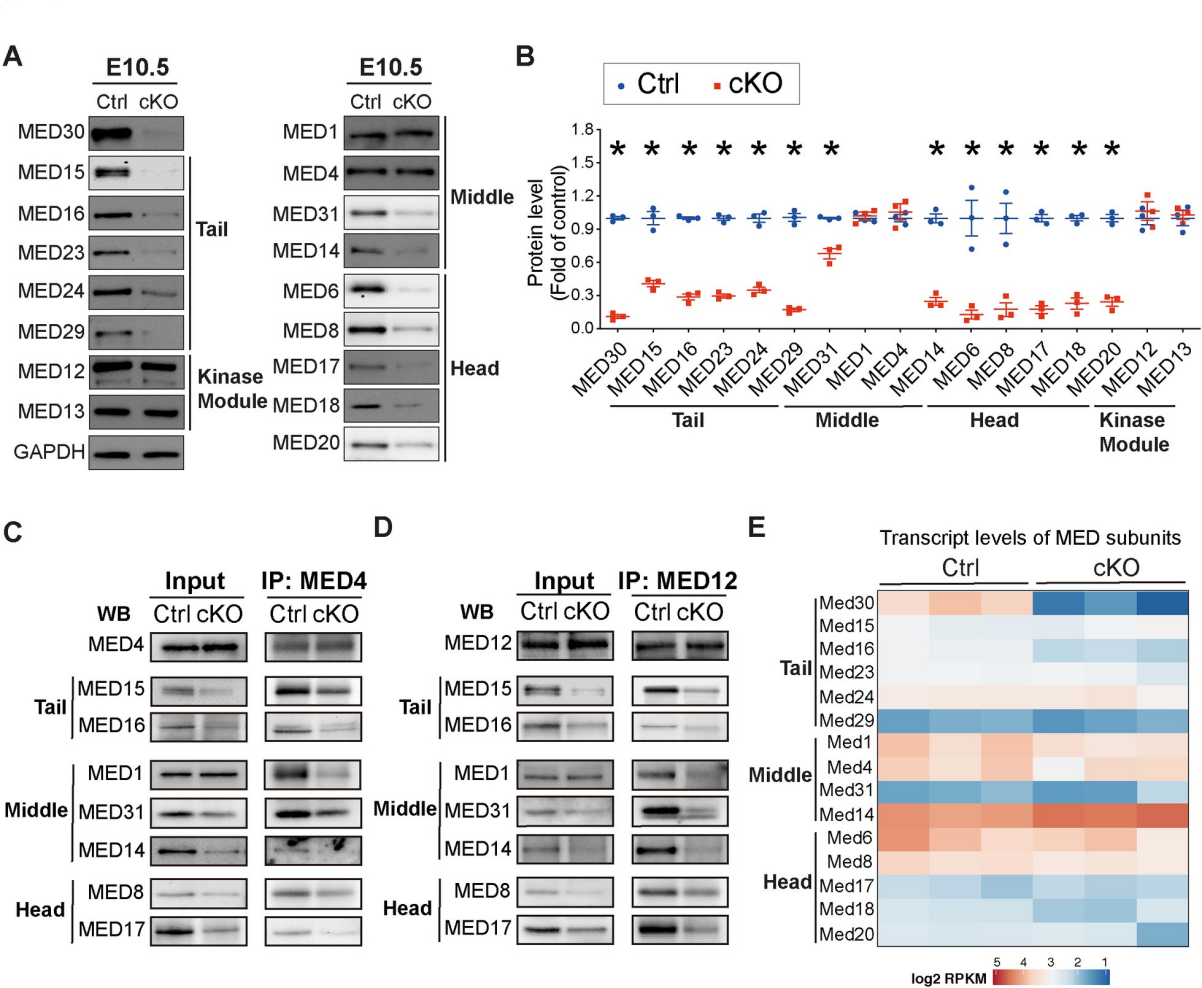
Loss of MED30 results in degradation of Mediator core in developing cardiomyocytes. **(A-B)** Representative immunoblots (A) and quantification analysis (B) of MED30 and MED Tail submodule subunits MED15, MED16, MED23, MED24 and MED29; MED Middle submodule subunits MED1, MED4, MED31 and MED14; MED Head submodule subunits MED6, MED8, MED17, MED18 and MED20, and Kinase module subunits MED12 and MED13 in hearts isolated from *Med30* cKO and control (Ctrl) embryos at E10.5. GAPDH served as a loading control. n = 3. **(C-D)** Co-Immunoprecipitation (Co-IP) of E10.5 heart lysates with antibody to MED4 (C) or MED12 (D), followed by Western blot (WB) analysis for MED subunits. 40 hearts from each genotypes were pooled for one IP experiment. **(E)** Heatmap representation of transcript levels of selected Mediator subunits in *Med30* cKO and Ctrl hears at E10.5. Data are represented as the mean ± SEM. *P < 0.05, by 2-tailed Student’s t test.

MED30 belongs to the Mediator core complex, which contains 26 subunits and consists of three sub-modules: the Tail, Middle and Head. To determine the molecular mechanism by which loss of MED30 in cardiomyocytes resulted in abnormal heart development leading to early embryonic lethality, we first assessed whether deletion of *Med30* in developing cardiomyocytes affected the integrity of the Mediator complex in cardiomyocytes. We performed western blot analysis to examine protein levels of Mediator subunits at E10.5. We examined representative subunits of each Mediator core submodule: MED15, MED16, MED23, MED24, MED29 within the Tail submodule; MED1, MED4, MED31, MED14 within the Middle submodule; and MED6, MED8, MED17, MED18, MED20 within the Head submodule. We also examined representative subunits of the kinase module, MED12 and MED13. This analysis revealed that most Mediator core subunits, with the exception of MED1 and MED4 within the middle submodule, were significantly decreased in *Med30* cKO hearts compared to those of control littermates at E10.5 (Fig 2A and 2B). MED4 displayed a significant decrease in *Med30* cKOs at E11.5, perhaps reflecting a slower rate of degradation, while MED1 remained unchanged at this stage (S3 Fig). However, the kinase module members MED12 and MED13 were not changed in cKO hearts at either E10.5 or E11.5, when compared to controls (Figs 2A–2B and S3). To further determine the role of MED30 in the stability of the Mediator core, we performed immunoprecipitation experiments from E10.5 MED30 cKO and control hearts utilizing antibodies to MED4 and MED12. Our results revealed that levels of MED core subunits were significantly decreased in MED complexes immunoprecipitated from MED30 cKO hearts compared to those from control hearts (Fig 2C and 2D). Notably, although total MED1 protein levels were similar in MED30 cKO and control hearts (Figs 2A–2B and S3), the level of MED1 present in MED4 and MED12 immunoprecipitates was nonetheless significantly reduced in cKO hearts, suggesting that the preserved MED1 was not associated with the Mediator complex. RNAseq analyses of embryonic hearts isolated from *Med30* cKOs and controls at E10.5 revealed that transcript levels of Mediator subunits we examined were not decreased in *Med30* cKOs compared to controls (Fig 2E). These results strongly suggest that MED30 is essential to maintain protein stability of core MED subunits, but neither expression nor stability of kinase module subunits.

### Transcriptome in developing heart regulated by Mediator complex

**Fig 3:**
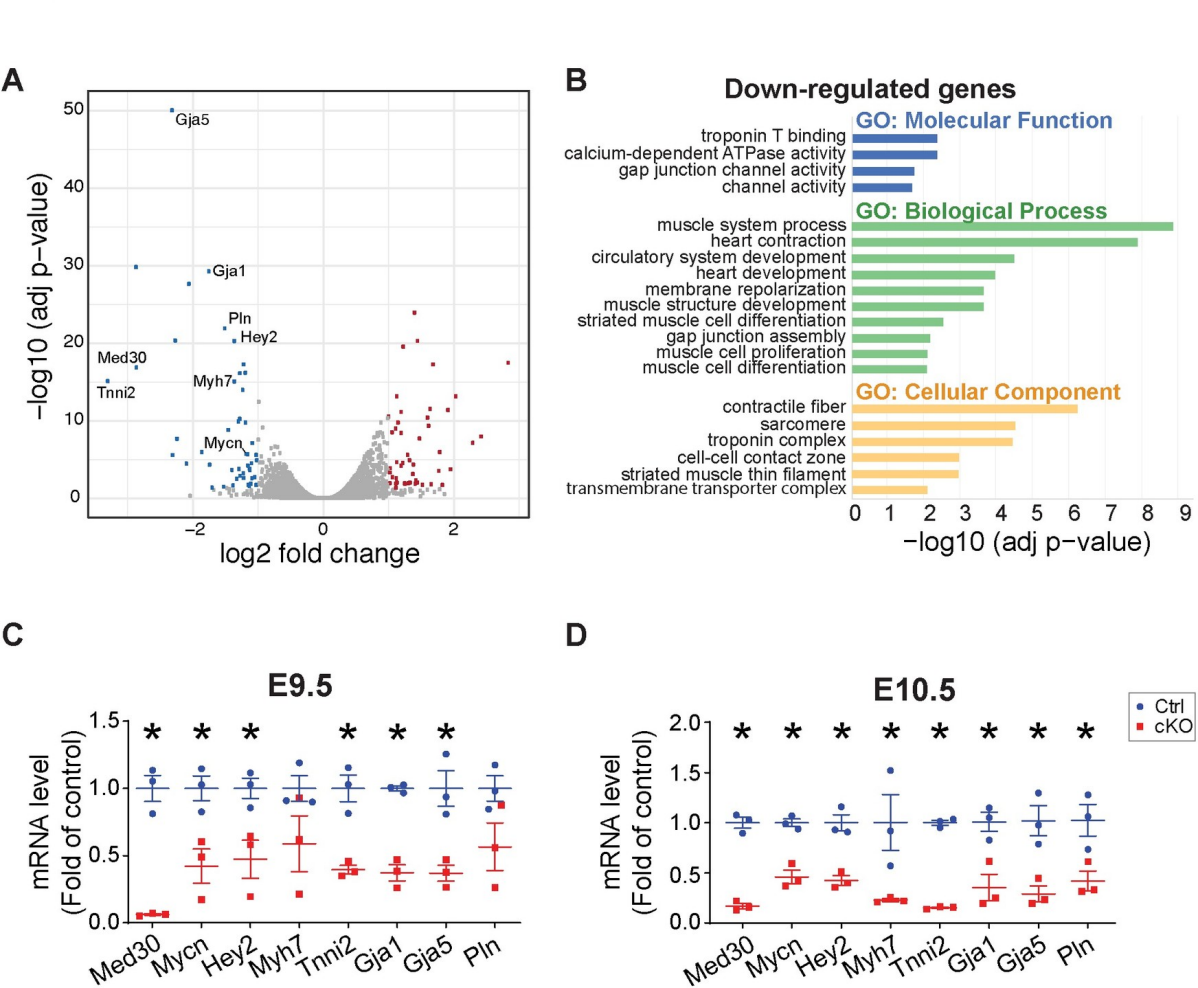
Transcriptome analysis in *Med30* cardiomyocyte-specific knockout (cKO) and control (Ctrl) hearts. (**A**) Volcano plot obtained from DESeq2 analysis of gene expression in *Med30* cKO versus control hearts at E10.5. Genes with adjusted P<0.05 and log2 (fold change) >1 are considered significantly upregulated or downregulated genes in *Med30* cKO hearts. The upregulated or downregulated genes are highlighted in red and blue, respectively. **(B)** Gene Ontology (GO) analysis of significantly downregulated genes in *Med30* cKO hearts. **(C, D)** qRT-PCR validation of RNA-seq data in *Med30* cKO (red) versus control (Ctrl) (blue) hearts at E9.5 (C) and E10.5 (D). Data were normalized to corresponding 18s levels, and cKO is expressed as the fold-change versus control. n = 3. Data are represented as the mean ± SEM. *P < 0.05, by 2-tailed Student’s t test.

The Mediator complex plays a central role in transcription initiation by integrating regulatory signals from gene-specific transcriptional activators to RNA polymerase II (Pol II) [8–12]. Because we observed that loss of MED30 resulted in destabilization of the Mediator core, our *Med30* cKO mouse model provided us with a unique opportunity to study the transcriptome in developing heart controlled by the intact Mediator complex. RNA-seq analysis was performed on RNA extracted from hearts of *Med30* cKO and control littermates at E10.5. From this analysis, 31 genes were significantly upregulated, and 45 genes were significantly downregulated (log2fold > 1, adjusted p value < 0.05) in mutant cardiomyocyte relative to controls (Fig 3A). Clustering of differentially expressed genes by molecular function using Gene Ontology (GO) analysis demonstrated that important molecular pathways related to cardiac development were decreased in cKO hearts, including “Troponin T binding”, “channel activity”, “heart contraction”, “heart development”, “muscle cell proliferation” and “muscle cell differentiation” (Fig 3B). Decreased genes showed enrichment for “contractile fiber”, “sarcomere” and “troponin complex” (Fig 3B), while increased genes did not display any functional enrichment. Some significantly downregulated genes were likely to account for observed *Med30* cKO phenotypes. An example of these was *Mycn*, which is essential for cardiomyocyte proliferation and cardiogenesis [38,39]. *Mycn* null mice arrest development at E9.5 and die between E10.5 and E11.5 with severely hypoplastic hearts, undivided thin-walled ventricles, and few trabeculae [39]. Another significantly downregulated gene in *Med30* cKO hearts was *Hey2*, which plays a pivotal role in left ventricular maturation [40]. In addition, *Tnni2*, *Mhy7*, *Gja1*, *Gaj5*, and *Pln* encode proteins that are essential for cardiomyocyte structure and contractility [41]. Decreased expression of these critical heart development genes was validated by performing quantitative PCR (qPCR) of mRNA obtained from E9.5 and E10.5 hearts of *Med30* cKOs and control littermates (Fig 3C and 3D). Taken together, these results suggest that the Mediator complex controls a specific transcriptional program that is critical for cardiac development.

### MED30 deletion in adult cardiomyocytes leads to rapid development of DCM and lethality

**Fig 4:**
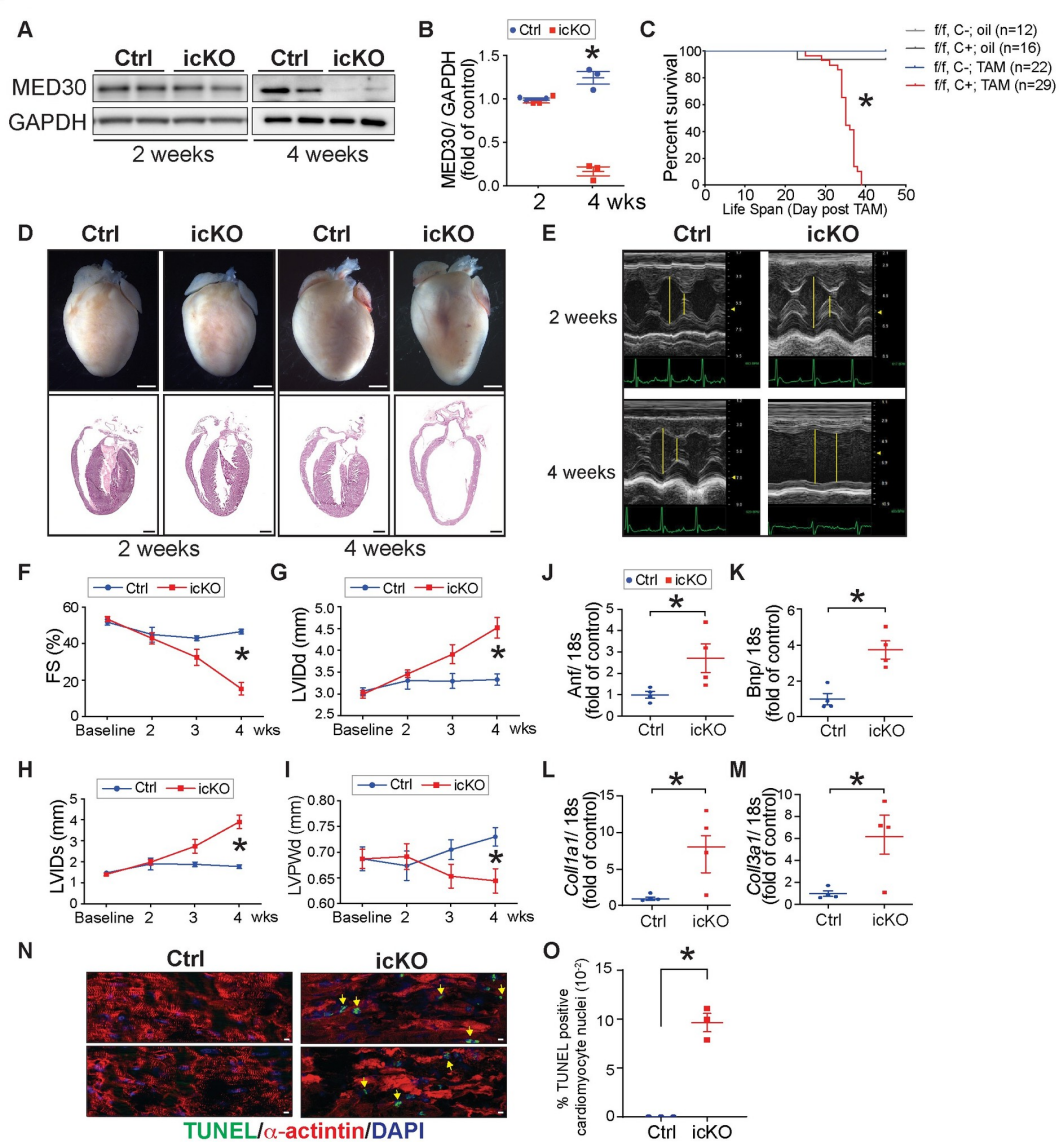
MED30 deletion in adult cardiomyocyte leads to rapidly developed dilated cardiomyopathy (DCM) and lethality. **(A-B)** Representative immunoblots (A) and quantification analysis (B) of MED30 in the heart isolated from inducible *Med30* cardiomyocyte specific knockout (icKO) and control (Ctrl) mice at 2 weeks and 4 weeks post-tamoxifen injection. GAPDH served as a loading control. n = 3. **(C)** Kaplan-Meier survival curves of *Med30* icKO and control and mice after oil (n = 12–16) or tamoxifen injection (n = 22–29). **(D)** Representative hearts (top) and H&E stained sections (bottom) of *Med30* icKO and Ctrl mice at 2 weeks and 4 weeks post-tamoxifen injection. **(E)** Representative M-mode echocardiography of *Med30* icKO and Ctrl mice at 2 weeks and 4 weeks post-tamoxifen injection. **(F-I)** Echocardiographic measurements for *Med30* icKO and Ctrl mice at baseline, 2, 3 and 4 weeks (wks) post-tamoxifen injection by (F) fractional shortening (FS), and left ventricular (LV) internal dimensions at (G) end-diastole (LVIDd) and (H) end-systole (LVIDs), as well as LV posterior wall thickness at the end-diastolic (LVPWd). n = 14 mice per group. **(J-M)** qRT-PCR analysis of cardiac fetal gene markers atrial natriuretic factor (Anf) (J) and B-type natriuretic peptide (Bnp) (K), and profibrotic genes, collagen α1 types I (*Col1a1*) (L) and III (*Col3a1*) (M), in control and icKO mouse hearts at 4 weeks post-tamoxifen injection. Data were normalized to corresponding 18s levels, and icKO is expressed as the fold-change versus control. n = 3. **(N-O)** Representative immunostaining images (N) and quantification analysis (O) of TUNEL staining (green) in heart sections from *Med30* icKO and Ctrl mice at 4 weeks post-tamoxifen injection, using an antibody against α-actinin as cardiomyocyte marker (red). DNA is stained with DAPI (blue). Scale bar: 10 μm. n = 3. Data are represented as the mean ± SEM. *P < 0.05, by 2-tailed Student’s t test, or by the Kaplan-Meier survival analysis.

The embryonic lethality of *Med30* cKO mice precluded studying the role of MED30 in adult cardiomyocytes. We therefore generated tamoxifen-inducible cardiac-specific knockout (icKO) mice by crossing *Med30* floxed mice (*Med30* ^f/f^) with αMHC-MerCreMer transgenic mice to allow for inducible deletion of *Med30* in adult cardiomyocytes upon tamoxifen injection [42]. To induce postnatal ablation of MED30 in adult cardiomyocytes, 8-week-old *Med30* ^f/f^; αMHC-MerCreMer (icKO) and *Med30* ^f/f^; αMHC-MerCreMer negative control mice were injected peritoneally with tamoxifen, or peanut oil as vehicle control. Injection of tamoxifen to αMHC-MerCreMer mice (without a floxed allele) did not induce mortality or cardiac dysfunction, comparable to results with control Cre negative mice, demonstrating that no cardiac toxicity was induced by our tamoxifen injection protocol and the αMHC-MerCreMer allele (S4A–S4E Fig). Western blot analysis confirmed significantly diminished protein levels of MED30 in cardiac tissue isolated from *Med30* icKO mice, compared to those of control Cre-negative *Med30* ^f/f^ hearts, at 4-weeks post tamoxifen injection (Fig 4A and 4B). At 2-weeks post tamoxifen injection, although mRNA levels of *Med30* were decreased about 90%, protein levels were preserved, suggesting that MED30 exhibits a long half-life at this age (Figs 4A and S5A). *Med30* icKO mice began to die at day 23 post tamoxifen (Fig 4C). In contrast to controls, no icKO mice survived longer than 40-days post tamoxifen (Fig 4C), demonstrating that MED30 in adult cardiomyocytes was required for survival. Morphological and histological analysis of *Med30* icKO and control hearts revealed ventricular chamber dilation and thinner ventricular walls in *Med30* icKO hearts at 4-weeks post tamoxifen injection, while no significant changes were observed at 2-weeks post tamoxifen injection (Fig 4D).

To assess cardiac function in *Med30* icKO mice, a comprehensive time course of cardiac physiological studies was performed in *Med30* icKO mice and littermate controls. Results revealed a time-dependent decrease in left ventricular (LV) systolic function (percentage of fractional shortening [FS]) in *Med30* icKO mice relative to controls (Fig 4E and 4F). Consistent with histological observations, loss of MED30 in adult cardiomyocytes resulted in LV chamber dilation, as evidenced by a significant increase in end-diastolic LV internal diameter (LVIDd) and end-systolic LV internal diameter (LVIDs) (Fig 4G and 4H), as well as decreased left ventricular posterior wall thickness at end-diastole (LVPWd) (Fig 4I). We did not observe changes in the ratio of left ventricle weight to body weight (LV/BW) or the ratio of left ventricle weight to tibial length (LV/TL) in *Med30* icKO mice, compared to control, at 4-weeks following tamoxifen injection (S5B–S5C Fig). Consistent with molecular evidence of cardiac stress [43], cardiac fetal gene markers atrial natriuretic factor (ANF) and B-type natriuretic peptide (BNP) were significantly increased in hearts of icKO mice at 4 weeks of age (Fig 4J and 4K), as were profibrotic genes Collagen α1 types I (*Col1a1*) and III (*Col3a1*) (Fig 4L and 4M). Although we did not observe cardiac fibrosis by Masson’s trichrome staining (S5D Fig), we observed significant activation of fibroblasts by immunofluorescence staining for periostin (S5E Fig), which is expressed exclusively by fibroblasts or cells that adopt a fibroblast-like phenotype after injury [44], suggesting activation of fibrosis, although collagen had not yet accumulated in myocardium of *Med30* icKO mice. An increased number of TUNEL positive cardiomyocytes was observed in icKO hearts relative to controls at 4-weeks post tamoxifen injection (Fig 4N and 4O), suggesting that loss of MED30 in adult cardiomyocytes caused cardiomyocyte death. Taken together, these results demonstrate that MED30 is essential for adult cardiomyocyte function and viability. Deletion of MED30 in adult cardiomyocytes leads to rapid development of DCM and lethality.

### Transcriptome profile in MED30 icKO hearts

**Fig 5:**
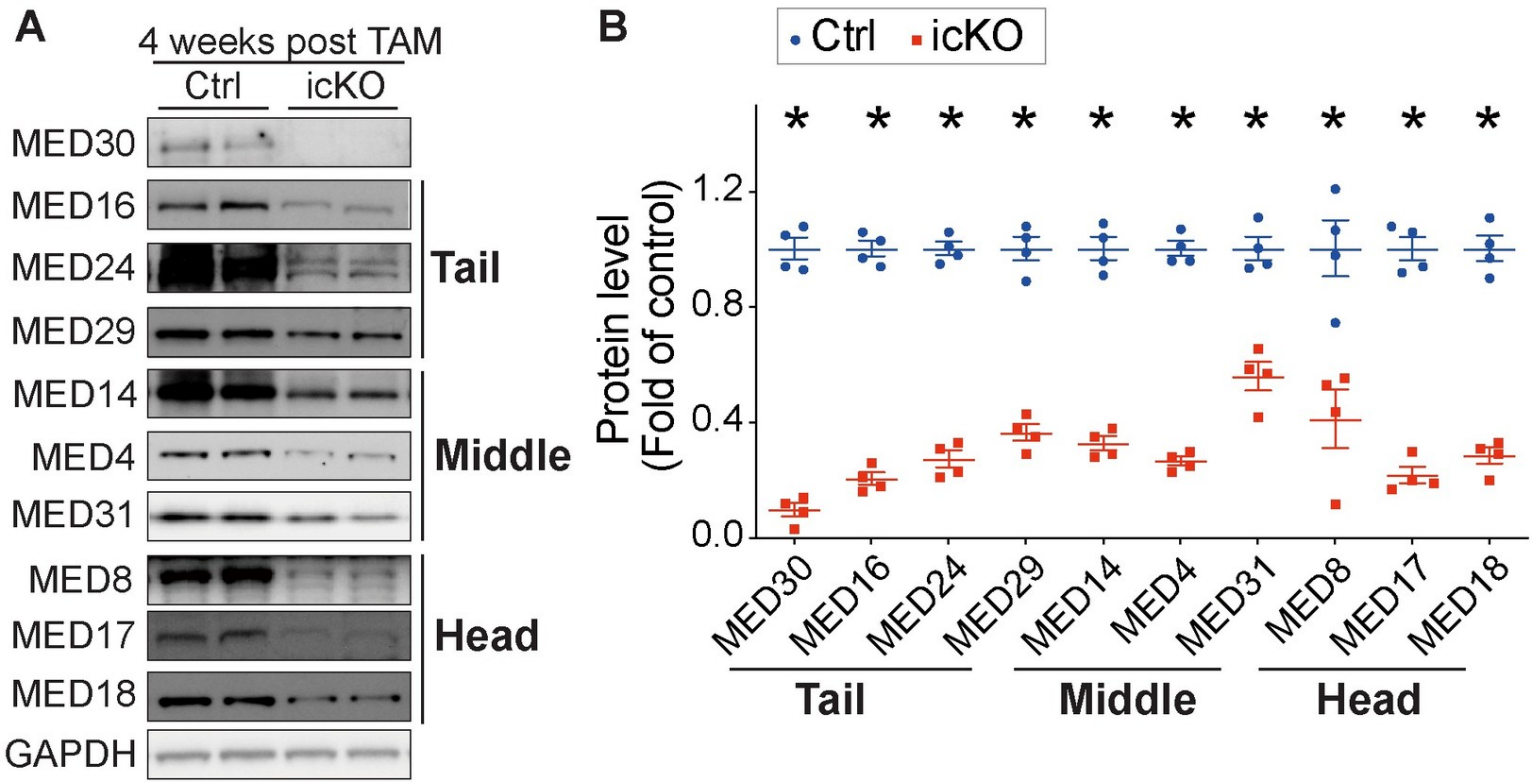
Loss of MED30 results in degradation of Mediator core in adult cardiomyocytes. **(A-B)** Representative immunoblots (A) and quantification analysis (B) of MED30 and MED Tail submodule subunits MED16, MED24, and MED29; MED Middle submodule subunits MED14, MED4 and MED31; MED Head submodule subunits MED8, MED17 and MED18 in adult cardiomyocytes isolated from *Med30* icKO and control (Ctrl) hearts at 4 weeks post-tamoxifen injection. GAPDH served as a loading control. n = 4. Data are represented as the mean ± SEM. *P < 0.05, by 2-tailed Student’s t test.

Our studies in developing cardiomyocytes suggested that MED30 was essential for the integrity of the Mediator core (Fig 2). To determine if this is also the case in adult cardiomyocytes, we performed western blot analysis of protein levels of Mediator core subunits in adult cardiomyocytes isolated from *Med30* icKO and control hearts at 4-weeks post tamoxifen injection. Consistent with results in *Med30* cKO hearts, we observed significant decreases in the protein levels of Mediator subunits comprising the Tail (MED16, MED24, MED29), Middle (MED4, MED31 and MED14), and Head (MED8, MED17 and MED18) submodules in *Med30* icKO cardiomyocytes, compared to controls (Fig 5A and 5B). This result suggests that the Mediator core is decreased in MED30 deficient cardiomyocytes. Accordingly, dysregulation of the transcriptome could contribute to the rapid progression of DCM in *Med30* icKO mice.

**Fig 6:**
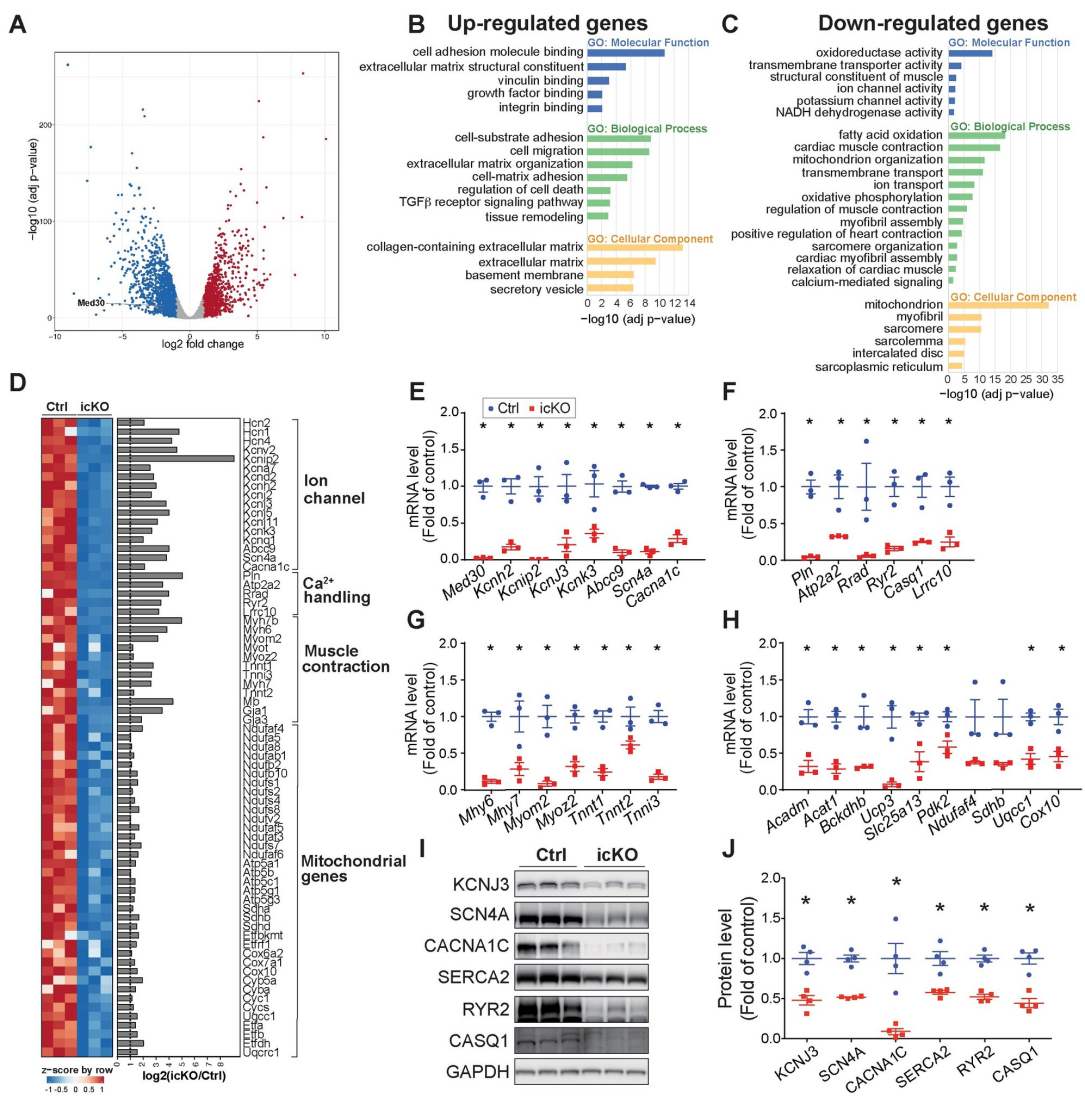
Transcriptome analysis in MED30 deletion adult cardiomyocytes. **(A)** Volcano plot obtained from DESeq2 analysis of gene expression in adult cardiomyocytes isolated from *Med30* icKO versus control hearts at 4 weeks post-tamoxifen injection. Genes with adjusted P<0.05 and log2 (fold change) >1 are considered significantly upregulated or downregulated genes in *Med30* icKO cardiomyocyte. The upregulated or downregulated genes are highlighted in red and blue, respectively. **(B-C)** Gene Ontology (GO) analysis of significantly upregulated (B) and downregulated (C) genes in cardiomyocytes isolated from *Med30* icKO at 4 weeks post-tamoxifen injection. **(D)** Heatmap (left) and bar graph of log2 (fold change) (right) for downregulated genes involved in ion channels, Ca^2+^ handling, muscle contraction and mitochondrial genes. **(E-H)** qRT-PCR validation of RNA-seq data for genes involved in ion channels (E), Ca^2+^ handling (F), muscle contraction (G), and mitochondrial genes (H), in adult cardiomyocytes isolated from *Med30* icKO (red) versus control (Ctrl) (blue) at at 4 weeks post-tamoxifen injection. n = 3. **(I, J)** Representative immunoblots (I) and quantification analysis (J) of KCNJ3, SCN4A, CACNA1C, SERCA2, RYR2 and CASQ1 in adult cardiomyocytes isolated from *Med30* icKO and control (Ctrl) hearts at 4 weeks post-tamoxifen injection. GAPDH served as a loading Ctrl. n = 3. All Data represent mean ± SEM. Statistical significance was based on student’s t test, *, P <0.05.

To identify genes responsible for the rapidly developed phenotype in *Med30* icKO mice, we performed RNA-seq analysis of RNA extracted from adult cardiomyocytes isolated from *Med30* icKO and control mice at 4-weeks post tamoxifen injection. From this analysis, 9517 genes were detected in both mutant and control cardiomyocytes. Of these, 1153 and 1509 were significantly (log2fold > 1, adjust p value < 0.05) up- or downregulated, respectively, in mutant cardiomyocyte relative to controls (Fig 6A). Comparative analysis revealed a significant overlap of down-regulated genes in *Med30* cKO hearts and *Med30* icKO cardiomyocytes (S6 Fig). Among genes that were decreased in cKO hearts, 24 of 45 were also decreased in adult cardiomyocytes with MED30 deletion (S6 Fig), suggesting that MED30 controls a common group of genes in both embryonic and adult cardiomyocytes. By contrast, 21 of 45 genes were uniquely decreased in *Med30* cKO hearts, but not in icKO cardiomyocytes, while 1485 out of 1509 genes were uniquely decreased in *Med30* icKO cardiomyocytes, but not in *Med30* cKO hearts. These results suggest MED30 also controls expression of specific genes in a stage-specific manner.

Clustering of differentially expressed genes by Gene Ontology (GO) gave an overview of known cellular pathways most severely altered in mutant cardiomyocytes. Results revealed that upregulated genes were enriched in pathways related to cardiac remodeling, fibrosis and cell death (Fig 6B), which could potentially explain our observation of increased cardiac fibroblast activation and elevated cell death in *Med30* icKO hearts. Consistent with previous results in *Med30* cKO hearts, some of the most significantly downregulated categories in icKO cardiomyocytes were associated with cardiac muscle contraction and ion channel activity (Fig 6C and 6D). Moreover, we observed a significant number of mitochondrial genes significantly decreased in MED30 deficient adult cardiomyocytes (Fig 6C and 6D), similar to observations with the *Med30* hypomorphic mutation that caused DCM in mice [36]. However, we note that fold-changes in ion channel, Ca^2+^ handling, and muscle contraction genes (log2 fold > 3), which directly control cardiac contractility, were more dramatic than fold-changes in mitochondrial genes (log2 fold <2) (Fig 6D). In particular, ion channel and Ca^2+^ handling genes were decreased by more than 90% (Fig 6D). RT-qPCR analyses of *Med30* icKO and control cardiomyocytes further validated RNAseq results (Fig 6E and 6H). We further performed western blot analysis to examine protein levels of KCNJ3, SCN4A, CACNA1C, SERCA2, RYR2 and CASQ1, which are critical for ion homeostasis and Ca^2+^ signaling, in adult cardiomyocytes isolated from *Med30* icKO and control mice at 4-weeks post tamoxifen injection. As shown in Fig 6I and 6J, these proteins were dramatically decreased in MED30 deficient cardiomyocytes.

Given that decreased mitochondrial gene expression in *Med30*^zg/zg^ mutants was postulated to account for observed DCM, as supported by prolongation of survival by several weeks using a ketogenic diet [36] we examined whether a ketogenic diet would rescue the lethality observed in *Med30* icKOs. *Med30* icKO and control mice were fed a ketogenic diet beginning at the time of tamoxifen injection. However, as shown in S7 Fig, *Med30* icKO mice displayed the same lethality on either the ketogenic diet or the control diet. Taken together, our findings demonstrate that MED30 is essential for the stability of the Mediator core and the normal transcriptional network in adult cardiomyocytes. Loss of MED30 in adult cardiomyocytes is catastrophic for heart function.

## Discussion

MED30 is associated with human congenital heart defects [29,34]. *Med30*^zg/zg^ mutant mice display DCM and postnatal lethality. However, the specific role of MED30 in cardiomyocytes is largely unknown. In this study, we generated a floxed *Med30* mouse line and used it to generate constitutive (cKO) and inducible (icKO) *Med30* cardiomyocyte-specific knockout mice. We have demonstrated that MED30 is essential for both developing and adult cardiomyocytes. Deletion of MED30 in cardiomyocytes caused rapid development of lethality in both embryonic and adult stages, with more severe phenotypes than that of *Med30*^zg/zg^ mutants, further supporting that the *Med30*^zg^ allele is a hypomorphic allele.

Loss of MED30 resulted in dramatically decreased proliferation in embryonic cardiomyocytes, and increased apoptosis in adult cardiomyocytes. Similar effects on cell cycle and cell viability have been observed in Med30 knockdown cancer cell lines [45,46]. MED30 is located within the Mediator core. While the assignment to a particular submodule was controversial due to lack to metazoan Mediator structural analysis [18], the newly revealed Mediator structure assigned MED30 to the proximal Tail and modeled de novo [19–23,35]. The small segment containing MED30, MED27, MED28, and MED29 forms proximal part of the Tail module and exhibits extensive interactions with the head submodule [19–23,35], suggesting a critical role for MED30 in the structure of the MED core. Our results demonstrated that loss of MED30 resulted in significantly decreased protein levels of multiple core Mediator subunits of the Head, Middle and Tail submodules, while the kinase module was preserved. These results strongly suggest that MED30 is essential to maintain protein stability of core Mediator subunits, but neither expression nor stability of kinase module subunits.

Our *Med30* cKO and icKO mouse models provided us with a unique opportunity to study the transcriptome in developing and adult heart controlled by the Mediator complex. In RNAseq results from both cKO hearts and icKO cardiomyocytes, we found that genes involved in muscle structure and contractility were significantly decreased. Consistent with results of others studying the hypomorphic *Med30*^zg/zg^ mutant, we also observed that genome-encoded mitochondrial genes were significantly decreased in MED30 deleted adult cardiomyocytes, although more dramatic decreases were observed in genes involved in ion channel, Ca^2+^ handling, and muscle contraction (log2 fold > 3). In *Med30*^zg/zg^ mutants, a ketogenic diet prolonged survival by several weeks, suggesting that decreased expression of mitochondrial genes, which causes the classic mitochondrial cardiomyopathy, contributes at least in part to the DCM of MED30 hypomorphic mutants. However, a ketogenic diet did not provide any beneficial effects on survival of our *Med30* icKO mice, suggesting that MED30 controls other aspects of the critical transcriptional network required for cardiomyocyte survival and function, in addition to mitochondrial genes. Notably, we observed key differences in transcriptome changes following deletion of MED30 between embryonic and adult cardiomyocytes. We observed a small subset of genes was changed in embryonic heart versus a larger subset of genes in adult cardiomyocytes following deletion of MED30. It is of relevance to note that deletion of individual MED subunits in B cell, T cell, and embryonic stem cells (ESC) revealed that only a fraction of genes was affected and that the number of differentially expressed genes was variable in different cell types, although the overall viability of cells was dramatically decreased [35]. Variation in the number of genes affected by MED subunit deletion has also been widely recognized in multiple other cell types, such as deletion of MED23 in mouse embryonic stem cells (ESCs) [47], mouse embryonic fibroblasts (MEFs) [48], mesenchymal stem cells [49], hematopoietic stem cells [50], adult hippocampal neural stem cells (NSCs) [51] and hepatocytes [52]. Another possible explanation for the large difference in the number of genes differentially expressed consequent to MED30 ablation in embryonic versus adult heart, is that, in embryonic heart, RNAseq was performed at E10.5, prior to onset of overt cardiac phenotypes, therefore likely excluding secondary gene changes. However, in adult hearts, RNAseq was performed 4 weeks post tamoxifen injection, as the very rapid onset of cardiomyopathy made it difficult to capture a time point prior to cardiac dysfunction. Thus, the differentially expressed genes observed in adult cardiomyocytes are likely to contain both primary targets and secondary changes that may reflect cardiac dysfunction. A comparison of our data from embryonic heart and adult cardiomyocytes revealed that MED30 controls a common group of genes in both embryonic and adult cardiomyocytes, but also controls expression of distinct subsets of genes in a stage-specific manner (S6 Fig). Genes regulated in a stage-specific manner may account for the distinct phenotypes observed at the two stages. For example, GO term analysis revealed that “muscle cell proliferation” genes were decreased in embryonic *Med30* mutant hearts (Fig 3B), while “regulation of cell death” genes were upregulated in adult *Med30* mutant cardiomyocytes (Fig 6B), consistent with phenotypic data showing proliferative defects in the embryo, and apoptosis in adult cardiomyocytes. Owing to lack of a suitable antibody for ChIP-seq analysis, it remains unknown whether the dysregulated genes we identified in *Med30* cKO hearts and icKO cardiomyocytes are direct or indirect targets of MED30.

Taken together, our study identifies an essential physiological role for MED30 in the stability of the Mediator core complex, and cardiac development and maintenance of normal cardiac morphology and function. Our results provide complementary evidence to cryo-EM analysis and reveal an important role of MED30 in Mediator stability.

## Materials and methods

### Ethical approval

The UCSD animal care personnel maintained all animals, and the IACUC of UCSD approved all experimental procedures. UCSD has an Animal Welfare Assurance document (A3033-01) on file with the Office of Laboratory Animal Welfare and is fully accredited by the Association for Assessment and Accreditation of Laboratory Animal Care (AAALAC) International.

### Mouse models

C57BL/6 mice (strain code: 027) were purchased from Charles River Laboratories. FLPase (FLP) mice [B6.SJL-Tg(ACT- FLPe)9205Dym/J, stock no. 005703] were purchased from The Jackson Laboratory. *Med30* floxed mice were generated by homologous recombination. The targeting vector was constructed using a plasmid containing a Neomycin cassette flanked by two Frt sites [53,54]. A 827-bp PCR product containing exon 1 of the mouse *Med30* gene was inserted into the BamHI site flanked by two loxP sites. A 5.2-kb upstream fragment and 3.8-kb downstream fragment were inserted into the vector to serve as 5′ and 3′ homologous arms, respectively. The completed targeting vector was linearized with NotI and electroporated into R1 mouse ES cells derived from 129-SV/J mice (University of California, San Diego Transgenic and Gene Targeting Core). After G418 selection, successfully targeted ES-cell clones were identified by Southern blot analysis after digestion with HindIII. The WT allele generated a 11.1-kb band, whereas the correctly targeted mutant allele generated a 6.5-kb band. One heterozygous recombinant ES clone was identified and microinjected into blastocysts from C57BL/6J mice to generate male chimeras. Male chimeras were bred with female C57BL/6J mice to generate germline-transmitted *Med30* floxed mice (*Med30* ^flox-neo/+^). *Med30* ^flox-neo/+^ mice were then crossed with FLP deleter mice [55] to remove the neomycin gene. *Med30* ^flox/+^ mutant mice were subsequently intercrossed to generate homozygous *Med30* ^flox/flox^ mice. To generate constitutive *Med30* cardiomyocyte-specific knockout (cKO) mice, *Med30* ^flox/flox^ mice were crossed with cardiac Troponin T (cTNT)-Cre transgenic mice [37,56]. The corresponding littermate controls were *Med30* ^flox/flox^ cTNT-Cre negative mice. To generate inducible *Med30* cardiomyocyte-specific knockout (icKO) mice, *Med30* ^flox/flox^ mice were crossed with α-MHC-MerCreMer transgenic mice [42]. Tamoxifen (TAM) (40 mg/kg/day for 3 days) was injected to induce gene ablation in adult cardiomyocytes [57]. Primer sequences for genotyping PCR are listed in S1 Table.

### Animal procedures and echocardiography

Echocardiography was performed as previously described [58,59]. Mice were briefly anesthetized with 1% isoflurane and underwent echocardiography using a FUJIFILM VisualSonics SonoSite Vevo 2100 ultrasound system with a 32- to 55-MHz linear transducer. The percentage of FS was used as an indicator of systolic cardiac function. Measurements of heart rate (HR), LVIDs, and LVIDd were determined from the LV M-mode tracing.

### Histological analysis

Mouse embryos or adult hearts were freshly dissected at various development stages and fixed in ice-cold phosphate buffered saline (PBS) with 4% paraformaldehyde overnight at 4°C. For cryosection, fixed tissues were incubated with concentrations of 5, 10, 15, and 20% sucrose solution diluted in PBS. Dehydrated tissues were embedded in OCT Tissue-Tek (Thermo Fisher Scientific), and cut into 8-μm sections using a Leica CM 3050S cryostat (Leica Microsystems). For paraffin section, after fixation, embryos were dehydrated with a series of 30–100% ethanol, embedded in paraffin, and cut into 8-μm sections by Microtome. Sections were stained with hematoxylin and eosin (H&E) or Masson Trichrome as previously described, and then mounted and imaged using a Hamamatsu NanoZoomer 2.0HT Slide Scanning System [53,60].

### Adult cardiomyocyte isolation

Adult cardiomyocytes were isolated as previously described [61]. Briefly, the heart was removed following isoflurane anesthesia and rinsed in Krebs-Henseleit buffer B (KHB- B) (118 mM NaCl, 4.8 mM KCl, 25 mM HEPES, 1.25 mM K2HPO4, 1.25 mM MgSO4, 11 mM glucose, pH 7.4). The heart was cannulated through the aorta and perfused on a Langendorff apparatus with KHB solution (3 ~ 5 minutes, 37°C), then incubated with KHB enzyme solution (0.5 mg/ml Collagenase Type 2, 0.5 mg/ml Collagenase Type 4, 25 μM Blebbistatin) for 12 minutes at 37°C. After digestion, the heart was perfused with 5 ml KHB solution to wash out the collagenase. The hearts were then minced in KHB solution with 2% BSA, gently agitated, and filtered through a 100μm polyethylene mesh. After settling, cells were centrifuged at 500 rpm at 4°C for 1 min. The pellet was resuspended in KHB solution and collected after centrifuged at 500 rpm at 4°C for 1 min again.

### RNA-seq data analysis

RNA-seq data were analyzed as previously described [61]. Briefly, RNA-seq reads were mapped against the reference mouse genome (GRCm38/mm10) for counting reads and gene expression. RPKM more than 1 was set as the threshold of removing genes with low counts. The DEseq2 v1.12.4 package (under R v3.4) was used to evaluate the reproducibility and perform differential gene expression analysis. Reads counts were normalized and a rlog transformation was applied for distance clustering and principal component analysis. Differentially expressed genes were detected by the default settings of DESeq2. We specified the apeglm method to shrink the log2 fold change, and the Benjamin-Hochberg algorithm to adjust p-value. Log2 fold changes > 1 or < -1 and adjusted p-value < 0.05 were then used to select differentially expressed genes. Gene list enrichment analyses were performed by using DAVID v6.8 (https://david.ncifcrf.gov/).

### Quantitative RT-PCR

Total RNA was extracted from embryonic mouse hearts or isolated cardiomyocytes using TRIzol reagent (Life Technologies, Thermo Fisher Scientific) according to the manufacturer’s recommendations. cDNA was synthesized using MMLV Reverse Transcriptase (Bio-Rad). Primer sequences for quantitative RT-PCR (qRT-PCR) are listed in S2 Table. RT PCR reactions were performed using Real-Time PCR Master Mix (Bio-Rad) in 96-well, low-profile PCR plates in a Bio-Rad CFX96 Thermocycler. Relative transcript abundance was normalized to 18s.

### Protein isolation and Western blot analysis

Total protein extracts were prepared as previously described [62]. Briefly, ground heart tissue or isolated cardiomyocytes were suspended in urea lysis buffer (8 M urea, 2 M thiourea, 3% SDS, 75 mM DTT, 0.03% bromophenol blue, 0.05 M Tris-HCl, pH 6.8). Protein lysates were separated on 4% to 12% SDS-PAGE gels (Life Technologies, Thermo Fisher Scientific) and transferred overnight at 4°C onto PVDF membranes (Bio-Rad). After blocking for 1 hour in TBS containing 0.1% Tween-20 (TBST) and 5% dry milk, the membranes were incubated overnight at 4°C with the indicated primary antibody (listed in S3 Table) in blocking buffer containing 2% dry milk. Blots were washed and incubated with HRP-conjugated secondary antibody generated in rabbit (1:3000) or mouse (1:3000) (Dako) for 1 hour at room temperature. Immunoreactive protein bands were visualized using the ECL reagent (Bio-Rad).

### Co-Immunoprecipitation (IP)

Heart tissue lysates from E10.5 *Med30* cKO or control mouse were rotated overnight at 4°C in 300 μL of IP lysis buffer (Thermo Scientific, 87788) with antibody after taking 5% lysates use for input. 50 μL of PBS-washed protein G magnetic beads (Thermo Scientific,88847) were resuspended after pre-wash 3 times by 0.02% PBST and incubated in lysate–antibody complexes for 4 hours at 4°C. After washing 3 times by using IP lysis buffer, beads were incubated with 100 μL 50mM pH2.8 Glycine, 4× LDS Sample loading buffer (Thermo Scientific, NP0007) and 10× Sample reducing agent (Thermo Scientific, NP0004) incubate with at 70°C for 10 minutes. The immunoprecipitates and input lysate were gel electrophoresed and immunoblotted.

### Immunostaining

Tissues were fixed and dehydrated, as described in the histological analysis. Sections were washed three times by PBS and blocked with block solution (1% BSA and 5% donkey serum in PBST (0.1% Triton in PBS)) for 1h at room temperature (RT). The sections were then incubated with primary antibody solution (1:200) (antibodies were added to block solution) overnight in a humidified chamber at 4°C. The following day, sections were washed three times with PBST and then incubated with secondary antibody (1:200) and DAPI (1:500) (antibodies were added in block solution) for 1.5h at RT. Primary and secondary antibodies are listed in S3 Table. EDU staining was performed by using the Click-i EdU Alexa Fluo 647 Imaging Kit (Invitrogen, Cat.C10340). TUNEL staining was performed by using *In Situ* Cell Death Detection Kit, Fluorescein (Sigma Aldrich, Cat. Roche-11684795910). The procedure followed the manufacturer’s instructions.

### Statistics

Data are presented as means ± SEM unless indicated otherwise. Statistical analysis was performed using GraphPad Prism 6.0 (GraphPad Software, La Jolla, CA) with a two-tailed Student’s *t*-test used for comparisons among groups. Survival data were calculated using Kaplan-Meier survival analysis with a log-rank statistical method. P values of <0.05 were considered statistically significant.

## Supporting information

S1 Fig Generation of *Med30* cardiomyocyte-specific knockout (cKO) mice. (A) Targeting strategy for the generation of *Med30* floxed mice. Neo, neomycin resistance gene; DTA, Diphtheria Toxin A chain gene. Green boxes abutted to the Neo gene indicate FRT sites. (B) Detection of wildtype (+) and targeted (m) alleles by Southern blot analysis. (C) Genotyping analysis for *Med30* floxed allele. (D-E) Representative immunoblots (D) and quantification analysis (E) of MED30 in hearts isolated from *Med30* cKO and control (Ctrl) mice at E9.5. GAPDH served as a loading control. n = 3. Data are represented as the mean ± SEM. *P < 0.05, by 2-tailed Student’s t test.
(TIF)

S2 Fig (Relative to Fig 1) MED30 is essential for early cardiac development. (A) Whole embryonic (top) and heart (middle) morphology (Scale bar: 1 mm), and H&E images (bottom) of *Med30* Ctrl and cKO littermates at E10.5 (Scale bar: 100 mm). n = 3. (B) Representative immunostaining images of TUNEL staining (green) in heart sections from *Med30* cKO and Ctrl mice at E11.5, using an antibody against α-actinin as cardiomyocyte marker (red). DNA is stained with DAPI (blue). Scale bar: 50 μm. n = 3.
(TIF)

S3 Fig (Relative to Fig 2) Loss of MED30 results in degradation of Mediator core in developing cardiomyocytes. (A-B) Representative immunoblots (A) and quantification analysis (B) of MED30, MED4, MED1, MED12, and MED13 in hearts isolated from *Med30* cKO (Blue) and Ctrl (control, Red) embryos at E11.5. GAPDH served as a loading control. n = 3. Data represent mean ± SEM. Statistical significance was based on student’s t test; *, P <0.05.
(TIF)

S4 Fig Injection of tamoxifen to α-MHC-MerCreMer dose not induced mortality or cardiac dysfunction. (A) Kaplan-Meier survival curves of α-MHC-MerCreMer positive (C+) (n = 9) and negative (C-) (n = 5) mice after tamoxifen (TAM) injection. (B-E) Echocardiographic measurements for C- and C+ mice at baseline and 4 weeks post-tamoxifen injection by (B) fractional shortening (FS), and left ventricular (LV) internal dimensions at (C) end-diastole (LVIDd) and (D) end-systole (LVIDs), as well as (E) LV posterior wall thickness at the end-diastolic (LVPWd). n = 5–9 mice per group. Data are represented as the mean ± SEM. *P < 0.05, by 2-tailed Student’s t test.
(TIF)

S5 Fig (Relative to Fig 4) MED30 deletion in adult cardiomyocytes lead to rapidly developed DCM and lethality. (A) qRT-PCR analysis of *Med30* in control (Ctrl) and icKO mouse hearts at 2 weeks post-tamoxifen injection. Data were normalized to corresponding 18s levels, and icKO is expressed as the fold-change versus control. n = 3. (B-C) Ratios of left ventricle weight to body weight (LV/BW) (B) and ratios of left ventricle weight to tibial length (LV/TL) (C) of control (Ctrl) and *Med30* icKO mice at 4 weeks post-tamoxifen injection, n = 8–11 mice per group. (D) Representative section views of Masson’s trichrome-staining of control (Ctrl) whole hearts (Top, left) and *Med30* icKO whole hearts (Top, middle) at 4 weeks post-tamoxifen injection isolated from mice, Masson’s trichrome positive control–stained kidney (Top, right), high-magnification images of the black box area (Bottom); scale bar: 1m (Top) and 50μm (Bottom). (E) Representative immunostaining images of Periostin staining (green) in heart sections from *Med30* icKO and Ctrl mice at 4 weeks post-tamoxifen injection, using an antibody against α-actinin as cardiomyocyte marker (red). DNA is stained with DAPI (blue). Scale bar: 50 μm. n = 3. Data represent mean ± SEM. Statistical significance was based on student’s t test; *, P <0.05.
(TIF)

S6 Fig The overlapping of *Med30* cKO and icKO down-regulated genes. Venn diagram shows overlapping genes between down-regulated genes in *Med30* cKO hearts (green) and icKO cardiomyocytes (brown). 24 overlapping genes were listed.
(TIF)

S7 Fig Ketogenic diet cannot rescue the lethality of *Med30* icKO mice. Kaplan-Meier survival curves of *Med30* icKO and control fed with either normal chow or ketogenic diet (KD). n = 11–14 mice per group.
(TIF)

S1 Table Genotyping primers. (PDF)

S2 Table List of qRT-PCR primers. (PDF)

S3 Table List of Antibodies. (PDF)

S4 Table Excel of numerical data used for graphs. (XLSX)

## References

[1] Bruneau BG. Signaling and transcriptional networks in heart development and regeneration. Cold Spring Harb Perspect Biol. 2013;5(3):a008292. doi: 10.1101/cshperspect.a008292 23457256 PMC3578359

[2] Nemer G, Nemer M. Regulation of heart development and function through combinatorial interactions of transcription factors. Ann Med. 2001;33(9):604–10. doi: 10.3109/07853890109002106 11817655

[3] Uosaki H, Cahan P, Lee DI, Wang S, Miyamoto M, Fernandez L, et al. Transcriptional Landscape of Cardiomyocyte Maturation. Cell Rep. 2015;13(8):1705–16. doi: 10.1016/j.celrep.2015.10.032 26586429 PMC4662925

[4] McCulley DJ, Black BL. Transcription factor pathways and congenital heart disease. Curr Top Dev Biol. 2012;100:253–77. doi: 10.1016/B978-0-12-387786-4.00008-7 22449847 PMC3684448

[5] Li X, Martinez-Fernandez A, Hartjes KA, Kocher JP, Olson TM, Terzic A, et al. Transcriptional atlas of cardiogenesis maps congenital heart disease interactome. Physiol Genomics. 2014;46(13):482–95. doi: 10.1152/physiolgenomics.00015.2014 24803680 PMC4080280

[6] Kohli S, Ahuja S, Rani V. Transcription factors in heart: promising therapeutic targets in cardiac hypertrophy. Curr Cardiol Rev. 2011;7(4):262–71. doi: 10.2174/157340311799960618 22758628 PMC3322445

[7] Akazawa H, Komuro I. Roles of cardiac transcription factors in cardiac hypertrophy. Circ Res. 2003;92(10):1079–88. doi: 10.1161/01.RES.0000072977.86706.23 12775656

[8] Malik S, Roeder RG. The metazoan Mediator co-activator complex as an integrative hub for transcriptional regulation. Nat Rev Genet. 2010;11(11):761–72. doi: 10.1038/nrg2901 20940737 PMC3217725

[9] Allen BL, Taatjes DJ. The Mediator complex: a central integrator of transcription. Nat Rev Mol Cell Biol. 2015;16(3):155–66. doi: 10.1038/nrm3951 25693131 PMC4963239

[10] Holstege FC, Jennings EG, Wyrick JJ, Lee TI, Hengartner CJ, Green MR, et al. Dissecting the regulatory circuitry of a eukaryotic genome. Cell. 1998;95(5):717–28. doi: 10.1016/s0092-8674(00)81641-4 9845373

[11] Myers LC, Gustafsson CM, Hayashibara KC, Brown PO, Kornberg RD. Mediator protein mutations that selectively abolish activated transcription. Proc Natl Acad Sci U S A. 1999;96(1):67–72. doi: 10.1073/pnas.96.1.67 9874773 PMC15094

[12] Kelleher RJ, 3rd, Flanagan PM, Kornberg RD. A novel mediator between activator proteins and the RNA polymerase II transcription apparatus. Cell. 1990;61(7):1209–15. doi: 10.1016/0092-8674(90)90685-8 2163759

[13] Bourbon HM. Comparative genomics supports a deep evolutionary origin for the large, four-module transcriptional mediator complex. Nucleic Acids Res. 2008;36(12):3993–4008. doi: 10.1093/nar/gkn349 18515835 PMC2475620

[14] Guglielmi B, van Berkum NL, Klapholz B, Bijma T, Boube M, Boschiero C, et al. A high resolution protein interaction map of the yeast Mediator complex. Nucleic Acids Res. 2004;32(18):5379–91. doi: 10.1093/nar/gkh878 15477388 PMC524289

[15] Cai G, Imasaki T, Takagi Y, Asturias FJ. Mediator structural conservation and implications for the regulation mechanism. Structure. 2009;17(4):559–67. doi: 10.1016/j.str.2009.01.016 19368889 PMC2673807

[16] Tsai KL, Tomomori-Sato C, Sato S, Conaway RC, Conaway JW, Asturias FJ. Subunit architecture and functional modular rearrangements of the transcriptional mediator complex. Cell. 2014;157(6):1430–44. doi: 10.1016/j.cell.2014.05.015 24882805 PMC4104964

[17] Wang X, Sun Q, Ding Z, Ji J, Wang J, Kong X, et al. Redefining the modular organization of the core Mediator complex. Cell Res. 2014;24(7):796–808. doi: 10.1038/cr.2014.64 24810298 PMC4085763

[18] Verger A, Monte D, Villeret V. Take Your PIC. Trends Biochem Sci. 2021. doi: 10.1016/j.tibs.2021.05.008 34103236

[19] Abdella R, Talyzina A, Chen S, Inouye CJ, Tjian R, He Y. Structure of the human Mediator-bound transcription preinitiation complex. Science. 2021;372(6537):52–6. doi: 10.1126/science.abg3074 33707221 PMC8117670

[20] Rengachari S, Schilbach S, Aibara S, Dienemann C, Cramer P. Structure of the human Mediator-RNA polymerase II pre-initiation complex. Nature. 2021;594(7861):129–33. doi: 10.1038/s41586-021-03555-7 33902108

[21] Chen X, Qi Y, Wu Z, Wang X, Li J, Zhao D, et al. Structural insights into preinitiation complex assembly on core promoters. Science. 2021;372(6541). doi: 10.1126/science.aba8490 33795473

[22] Chen X, Yin X, Li J, Wu Z, Qi Y, Wang X, et al. Structures of the human Mediator and Mediator-bound preinitiation complex. Science. 2021;372(6546). doi: 10.1126/science.abg0635 33958484

[23] Zhao H, Young N, Kalchschmidt J, Lieberman J, El Khattabi L, Casellas R, et al. Structure of mammalian Mediator complex reveals Tail module architecture and interaction with a conserved core. Nat Commun. 2021;12(1):1355. doi: 10.1038/s41467-021-21601-w 33649303 PMC7921410

[24] Berti L, Mittler G, Przemeck GK, Stelzer G, Gunzler B, Amati F, et al. Isolation and characterization of a novel gene from the DiGeorge chromosomal region that encodes for a mediator subunit. Genomics. 2001;74(3):320–32. doi: 10.1006/geno.2001.6566 11414760

[25] Muncke N, Jung C, Rudiger H, Ulmer H, Roeth R, Hubert A, et al. Missense mutations and gene interruption in PROSIT240, a novel TRAP240-like gene, in patients with congenital heart defect (transposition of the great arteries). Circulation. 2003;108(23):2843–50. doi: 10.1161/01.CIR.0000103684.77636.CD 14638541

[26] Asadollahi R, Oneda B, Sheth F, Azzarello-Burri S, Baldinger R, Joset P, et al. Dosage changes of MED13L further delineate its role in congenital heart defects and intellectual disability. Eur J Hum Genet. 2013;21(10):1100–4. doi: 10.1038/ejhg.2013.17 23403903 PMC3778355

[27] Chen CP, Chen YY, Chern SR, Wu PS, Su JW, Chen YT, et al. Prenatal diagnosis and molecular cytogenetic characterization of de novo partial trisomy 12q (12q24.21—>qter) and partial monosomy 6q (6q27—>qter) associated with coarctation of the aorta, ventriculomegaly and thickened nuchal fold. Gene. 2013;516(1):138–42. doi: 10.1016/j.gene.2012.12.051 23266815

[28] Schiano C, Casamassimi A, Vietri MT, Rienzo M, Napoli C. The roles of mediator complex in cardiovascular diseases. Biochim Biophys Acta. 2014;1839(6):444–51. doi: 10.1016/j.bbagrm.2014.04.012 24751643

[29] Napoli C, Schiano C, Soricelli A. Increasing evidence of pathogenic role of the Mediator (MED) complex in the development of cardiovascular diseases. Biochimie. 2019;165:1–8. doi: 10.1016/j.biochi.2019.06.014 31255603

[30] Trehan A, Brady JM, Maduro V, Bone WP, Huang Y, Golas GA, et al. MED23-associated intellectual disability in a non-consanguineous family. Am J Med Genet A. 2015;167(6):1374–80. doi: 10.1002/ajmg.a.37047 25845469 PMC5671761

[31] Au Yeung SL, Lin SL, Lam HS, Schooling CM. Effect of l-arginine, asymmetric dimethylarginine, and symmetric dimethylarginine on ischemic heart disease risk: A Mendelian randomization study. Am Heart J. 2016;182:54–61. doi: 10.1016/j.ahj.2016.07.021 27914500

[32] Basel-Vanagaite L, Smirin-Yosef P, Essakow JL, Tzur S, Lagovsky I, Maya I, et al. Homozygous MED25 mutation implicated in eye-intellectual disability syndrome. Hum Genet. 2015;134(6):577–87. doi: 10.1007/s00439-015-1541-x 25792360

[33] Leal A, Morera B, Del Valle G, Heuss D, Kayser C, Berghoff M, et al. A second locus for an axonal form of autosomal recessive Charcot-Marie-Tooth disease maps to chromosome 19q13.3. Am J Hum Genet. 2001;68(1):269–74. doi: 10.1086/316934 11112660 PMC1234926

[34] Chen CP, Lin MH, Chen YY, Chern SR, Chen YN, Wu PS, et al. Prenatal diagnosis and array comparative genomic hybridization characterization of interstitial deletions of 8q23.3-q24.11 and 8q24.13 associated with Langer-Giedion syndrome, Cornelia de Lange syndrome and haploinsufficiency of TRPS1, RAD21 and EXT1. Taiwan J Obstet Gynecol. 2015;54(5):592–6. doi: 10.1016/j.tjog.2015.08.013 26522117

[35] El Khattabi L, Zhao H, Kalchschmidt J, Young N, Jung S, Van Blerkom P, et al. A Pliable Mediator Acts as a Functional Rather Than an Architectural Bridge between Promoters and Enhancers. Cell. 2019;178(5):1145–58 e20. doi: 10.1016/j.cell.2019.07.011 31402173 PMC7533040

[36] Krebs P, Fan W, Chen YH, Tobita K, Downes MR, Wood MR, et al. Lethal mitochondrial cardiomyopathy in a hypomorphic Med30 mouse mutant is ameliorated by ketogenic diet. Proc Natl Acad Sci U S A. 2011;108(49):19678–82. doi: 10.1073/pnas.1117835108 22106289 PMC3241770

[37] Jiao K, Kulessa H, Tompkins K, Zhou Y, Batts L, Baldwin HS, et al. An essential role of Bmp4 in the atrioventricular septation of the mouse heart. Genes Dev. 2003;17(19):2362–7. doi: 10.1101/gad.1124803 12975322 PMC218073

[38] Charron J, Malynn BA, Fisher P, Stewart V, Jeannotte L, Goff SP, et al. Embryonic lethality in mice homozygous for a targeted disruption of the N-myc gene. Genes Dev. 1992;6(12A):2248–57. doi: 10.1101/gad.6.12a.2248 1459450

[39] Harmelink C, Peng Y, DeBenedittis P, Chen H, Shou W, Jiao K. Myocardial Mycn is essential for mouse ventricular wall morphogenesis. Dev Biol. 2013;373(1):53–63. doi: 10.1016/j.ydbio.2012.10.005 23063798 PMC3508168

[40] Koibuchi N, Chin MT. CHF1/Hey2 plays a pivotal role in left ventricular maturation through suppression of ectopic atrial gene expression. Circ Res. 2007;100(6):850–5. doi: 10.1161/01.RES.0000261693.13269.bf 17332425

[41] Sheng JJ, Jin JP. TNNI1, TNNI2 and TNNI3: Evolution, regulation, and protein structure-function relationships. Gene. 2016;576(1 Pt 3):385–94. 26526134 10.1016/j.gene.2015.10.052 PMC5798203

[42] Sohal DS, Nghiem M, Crackower MA, Witt SA, Kimball TR, Tymitz KM, et al. Temporally regulated and tissue-specific gene manipulations in the adult and embryonic heart using a tamoxifen-inducible Cre protein. Circ Res. 2001;89(1):20–5. doi: 10.1161/hh1301.092687 11440973

[43] Heineke J, Molkentin JD. Regulation of cardiac hypertrophy by intracellular signalling pathways. Nat Rev Mol Cell Biol. 2006;7(8):589–600. doi: 10.1038/nrm1983 16936699

[44] Moore-Morris T, Guimaraes-Camboa N, Yutzey KE, Puceat M, Evans SM. Cardiac fibroblasts: from development to heart failure. J Mol Med (Berl). 2015;93(8):823–30. doi: 10.1007/s00109-015-1314-y 26169532 PMC4512919

[45] Syring I, Weiten R, Muller T, Schmidt D, Steiner S, Kristiansen G, et al. The Contrasting Role of the Mediator Subunit MED30 in the Progression of Bladder Cancer. Anticancer Res. 2017;37(12):6685–95. doi: 10.21873/anticanres.12127 29187445

[46] Syring I, Weiten R, Muller T, Schmidt D, Steiner S, Muller SC, et al. The knockdown of the mediator complex subunit MED30 suppresses the proliferation and migration of renal cell carcinoma cells. Ann Diagn Pathol. 2018;34:18–26. doi: 10.1016/j.anndiagpath.2017.12.008 29661722

[47] Zhu W, Yao X, Liang Y, Liang D, Song L, Jing N, et al. Mediator Med23 deficiency enhances neural differentiation of murine embryonic stem cells through modulating BMP signaling. Development. 2015;142(3):465–76. doi: 10.1242/dev.112946 25564654

[48] Yin JW, Liang Y, Park JY, Chen D, Yao X, Xiao Q, et al. Mediator MED23 plays opposing roles in directing smooth muscle cell and adipocyte differentiation. Genes Dev. 2012;26(19):2192–205. doi: 10.1101/gad.192666.112 22972934 PMC3465740

[49] Liu Z, Yao X, Yan G, Xu Y, Yan J, Zou W, et al. Mediator MED23 cooperates with RUNX2 to drive osteoblast differentiation and bone development. Nat Commun. 2016;7:11149. doi: 10.1038/ncomms11149 27033977 PMC4821994

[50] Chen X, Zhao J, Gu C, Cui Y, Dai Y, Song G, et al. Med23 serves as a gatekeeper of the myeloid potential of hematopoietic stem cells. Nat Commun. 2018;9(1):3746. doi: 10.1038/s41467-018-06282-2 30218073 PMC6138688

[51] Chen GY, Zhang S, Li CH, Qi CC, Wang YZ, Chen JY, et al. Mediator Med23 Regulates Adult Hippocampal Neurogenesis. Front Cell Dev Biol. 2020;8:699. doi: 10.3389/fcell.2020.00699 32850819 PMC7403405

[52] Wang Z, Cao D, Li C, Min L, Wang G. Mediator MED23 regulates inflammatory responses and liver fibrosis. PLoS Biol. 2019;17(12):e3000563. doi: 10.1371/journal.pbio.3000563 31805036 PMC6917294

[53] Liang X, Zhou Q, Li X, Sun Y, Lu M, Dalton N, et al. PINCH1 plays an essential role in early murine embryonic development but is dispensable in ventricular cardiomyocytes. Mol Cell Biol. 2005;25(8):3056–62. doi: 10.1128/MCB.25.8.3056-3062.2005 15798193 PMC1069610

[54] Wu T, Mu Y, Bogomolovas J, Fang X, Veevers J, Nowak RB, et al. HSPB7 is indispensable for heart development by modulating actin filament assembly. Proc Natl Acad Sci U S A. 2017;114(45):11956–61. doi: 10.1073/pnas.1713763114 29078393 PMC5692592

[55] Rodriguez CI, Buchholz F, Galloway J, Sequerra R, Kasper J, Ayala R, et al. High-efficiency deleter mice show that FLPe is an alternative to Cre-loxP. Nat Genet. 2000;25(2):139–40. doi: 10.1038/75973 10835623

[56] Hirai M, Arita Y, McGlade CJ, Lee KF, Chen J, Evans SM. Adaptor proteins NUMB and NUMBL promote cell cycle withdrawal by targeting ERBB2 for degradation. J Clin Invest. 2017;127(2):569–82. doi: 10.1172/JCI91081 28067668 PMC5272190

[57] Zhou Y, Chen Z, Zhang L, Zhu M, Tan C, Zhou X, et al. Loss of Filamin C Is Catastrophic for Heart Function. Circulation. 2020;141(10):869–71. doi: 10.1161/CIRCULATIONAHA.119.044061 32150467 PMC7583669

[58] Anderson DJ, Kaplan DI, Bell KM, Koutsis K, Haynes JM, Mills RJ, et al. NKX2-5 regulates human cardiomyogenesis via a HEY2 dependent transcriptional network. Nat Commun. 2018;9(1):1373. doi: 10.1038/s41467-018-03714-x 29636455 PMC5893543

[59] Zhang Z, Mu Y, Veevers J, Peter AK, Manso AM, Bradford WH, et al. Postnatal Loss of Kindlin-2 Leads to Progressive Heart Failure. Circ Heart Fail. 2016;9(8). doi: 10.1161/CIRCHEARTFAILURE.116.003129 27502369 PMC4979611

[60] Zheng M, Cheng H, Li X, Zhang J, Cui L, Ouyang K, et al. Cardiac-specific ablation of Cypher leads to a severe form of dilated cardiomyopathy with premature death. Hum Mol Genet. 2009;18(4):701–13. doi: 10.1093/hmg/ddn400 19028670 PMC2722217

[61] Liu C, Spinozzi S, Chen JY, Fang X, Feng W, Perkins G, et al. Nexilin Is a New Component of Junctional Membrane Complexes Required for Cardiac T-Tubule Formation. Circulation. 2019;140(1):55–66. doi: 10.1161/CIRCULATIONAHA.119.039751 30982350 PMC6889818

[62] Fang X, Bogomolovas J, Wu T, Zhang W, Liu C, Veevers J, et al. Loss-of-function mutations in co-chaperone BAG3 destabilize small HSPs and cause cardiomyopathy. J Clin Invest. 2017;127(8):3189–200. doi: 10.1172/JCI94310 28737513 PMC5531406

