# Supplementary figures and images for: Mediator complex proximal Tail subunit MED30 is critical for Mediator core stability and cardiomyocyte transcriptional network
Source: PLoS Genet. 2021 Sep 10;17(9):e1009785. doi: 10.1371/journal.pgen.1009785 (PMC8432849; doi:10.1371/journal.pgen.1009785)

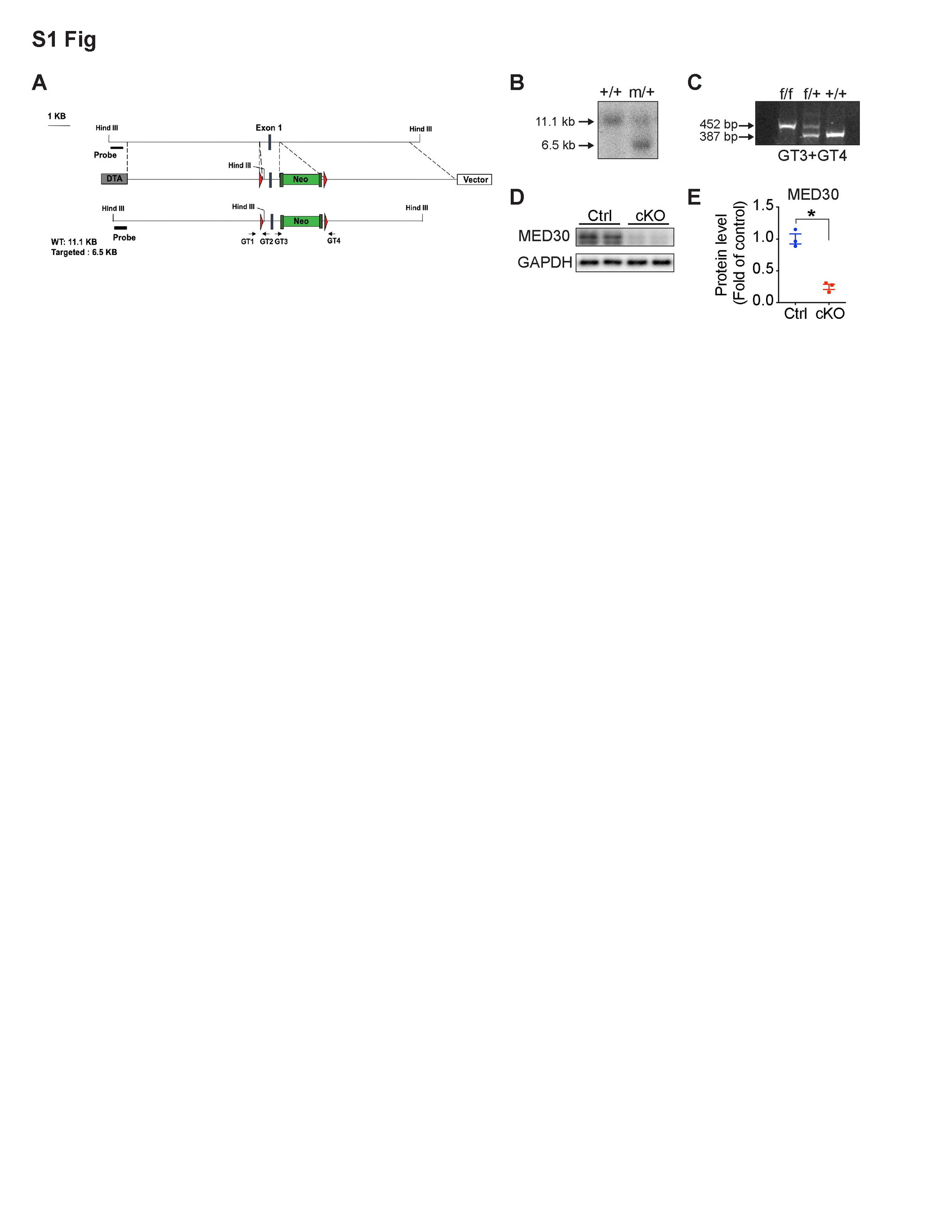

Supplement: S1 Fig — (A) Targeting strategy for the generation of Med30 floxed mice. Neo, neomycin resistance gene; DTA, Diphtheria Toxin A chain gene. Green boxes abutted to the Neo gene indicate FRT sites. (B) Detection of wildtype (+) and targeted (m) alleles by Southern blot analysis. (C) Genotyping analysis for Med30 floxed allele. (D-E) Representative immunoblots (D) and quantification analysis (E) of MED30 in hearts isolated from Med30 cKO and control (Ctrl) mice at E9.5. GAPDH served as a loading control. n = 3. Data are represented as the mean ± SEM. *P < 0.05, by 2-tailed Student’s t test. (TIF) [file pgen.1009785.s001.tif]

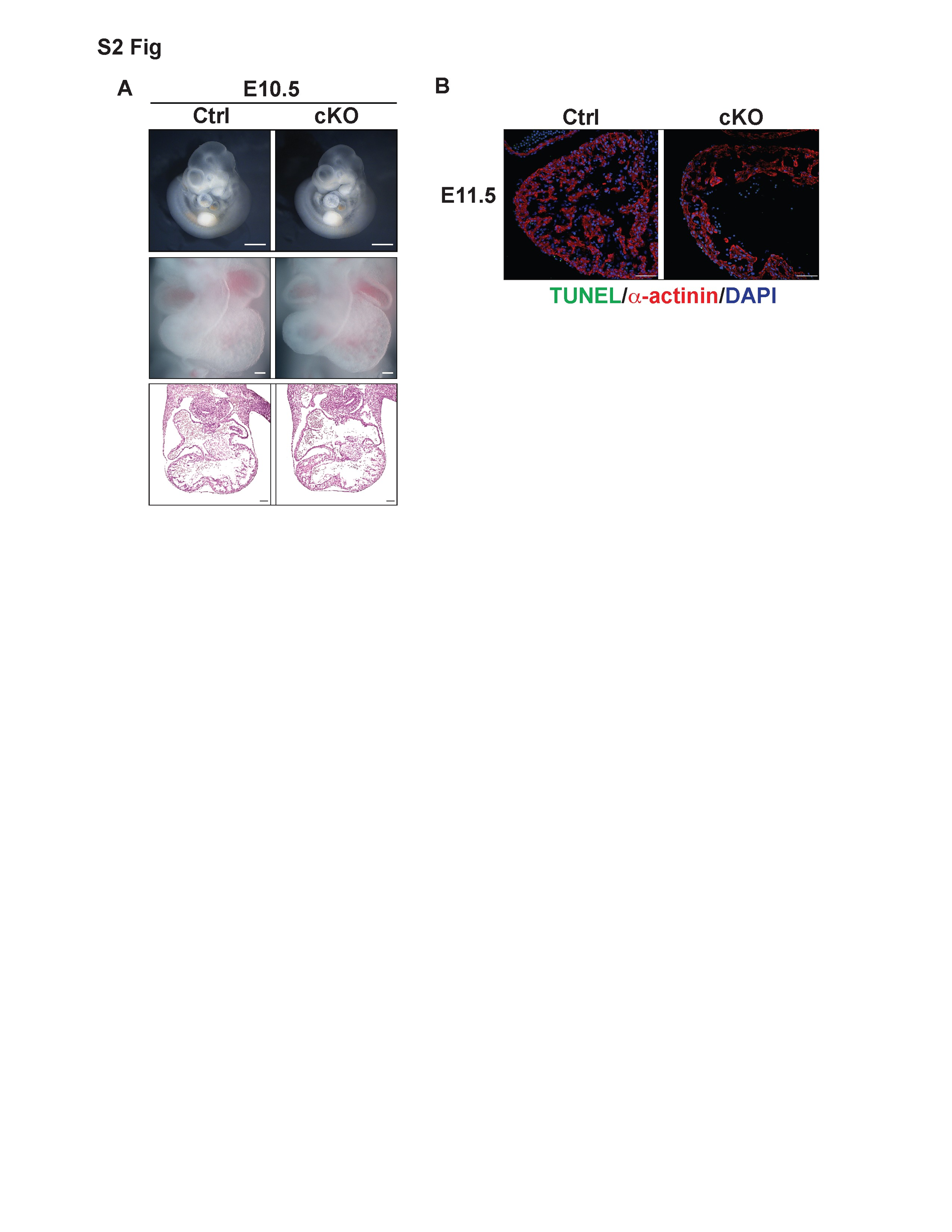

Supplement: S2 Fig — (A) Whole embryonic (top) and heart (middle) morphology (Scale bar: 1 mm), and H&E images (bottom) of Med30 Ctrl and cKO littermates at E10.5 (Scale bar: 100 mm). n = 3. (B) Representative immunostaining images of TUNEL staining (green) in heart sections from Med30 cKO and Ctrl mice at E11.5, using an antibody against α-actinin as cardiomyocyte marker (red). DNA is stained with DAPI (blue). Scale bar: 50 μm. n = 3. (TIF) [file pgen.1009785.s002.tif]

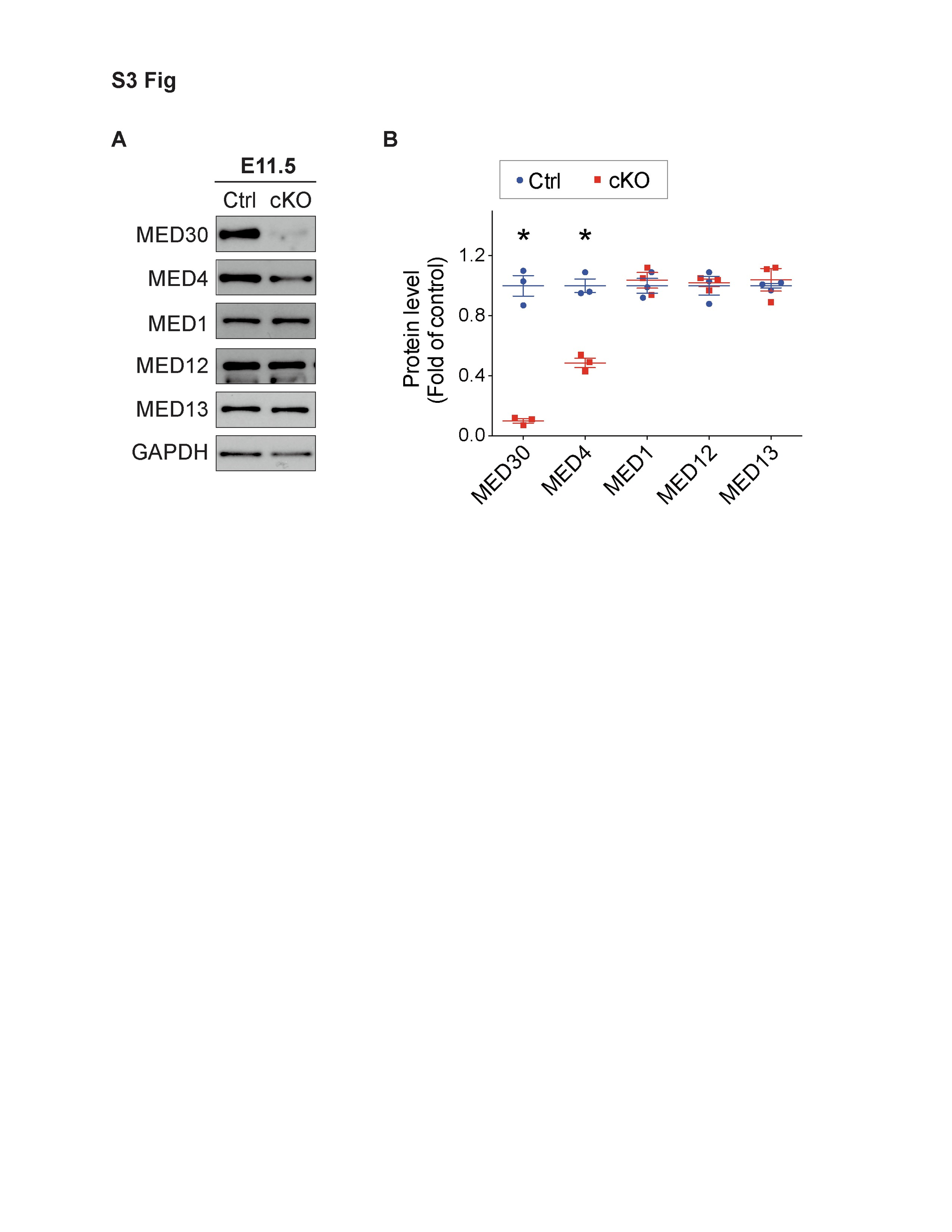

Supplement: S3 Fig — (A-B) Representative immunoblots (A) and quantification analysis (B) of MED30, MED4, MED1, MED12, and MED13 in hearts isolated from Med30 cKO (Blue) and Ctrl (control, Red) embryos at E11.5. GAPDH served as a loading control. n = 3. Data represent mean ± SEM. Statistical significance was based on student’s t test; *, P <0.05. (TIF) [file pgen.1009785.s003.tif]

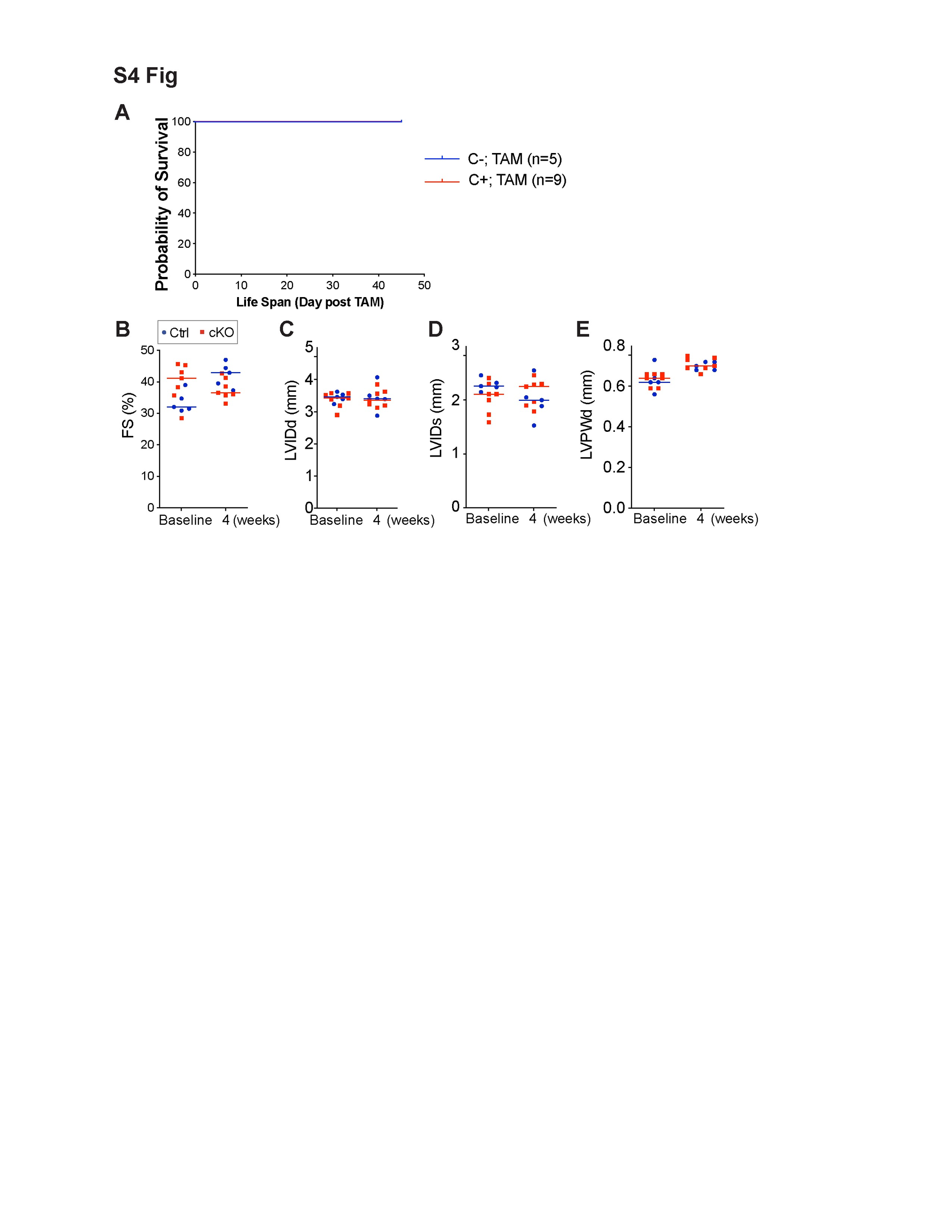

Supplement: S4 Fig — (A) Kaplan-Meier survival curves of α-MHC-MerCreMer positive (C+) (n = 9) and negative (C-) (n = 5) mice after tamoxifen (TAM) injection. (B-E) Echocardiographic measurements for C- and C+ mice at baseline and 4 weeks post-tamoxifen injection by (B) fractional shortening (FS), and left ventricular (LV) internal dimensions at (C) end-diastole (LVIDd) and (D) end-systole (LVIDs), as well as (E) LV posterior wall thickness at the end-diastolic (LVPWd). n = 5–9 mice per group. Data are represented as the mean ± SEM. *P < 0.05, by 2-tailed Student’s t test. (TIF) [file pgen.1009785.s004.tif]

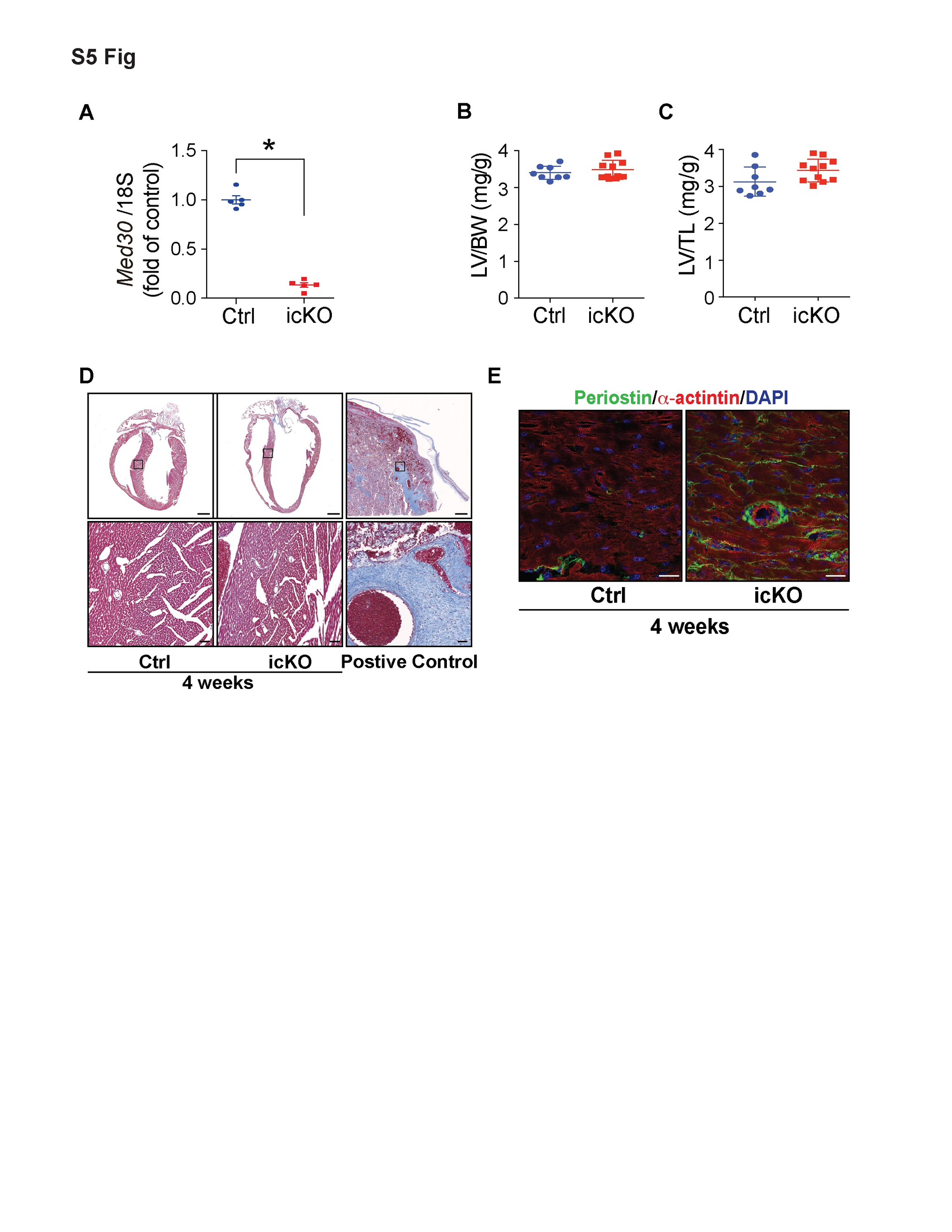

Supplement: S5 Fig — (A) qRT-PCR analysis of Med30 in control (Ctrl) and icKO mouse hearts at 2 weeks post-tamoxifen injection. Data were normalized to corresponding 18s levels, and icKO is expressed as the fold-change versus control. n = 3. (B-C) Ratios of left ventricle weight to body weight (LV/BW) (B) and ratios of left ventricle weight to tibial length (LV/TL) (C) of control (Ctrl) and Med30 icKO mice at 4 weeks post-tamoxifen injection, n = 8–11 mice per group. (D) Representative section views of Masson’s trichrome-staining of control (Ctrl) whole hearts (Top, left) and Med30 icKO whole hearts (Top, middle) at 4 weeks post-tamoxifen injection isolated from mice, Masson’s trichrome positive control–stained kidney (Top, right), high-magnification images of the black box area (Bottom); scale bar: 1m (Top) and 50μm (Bottom). (E) Representative immunostaining images of Periostin staining (green) in heart sections from Med30 icKO and Ctrl mice at 4 weeks post-tamoxifen injection, using an antibody against α-actinin as cardiomyocyte marker (red). DNA is stained with DAPI (blue). Scale bar: 50 μm. n = 3. Data represent mean ± SEM. Statistical significance was based on student’s t test; *, P <0.05. (TIF) [file pgen.1009785.s005.tif]

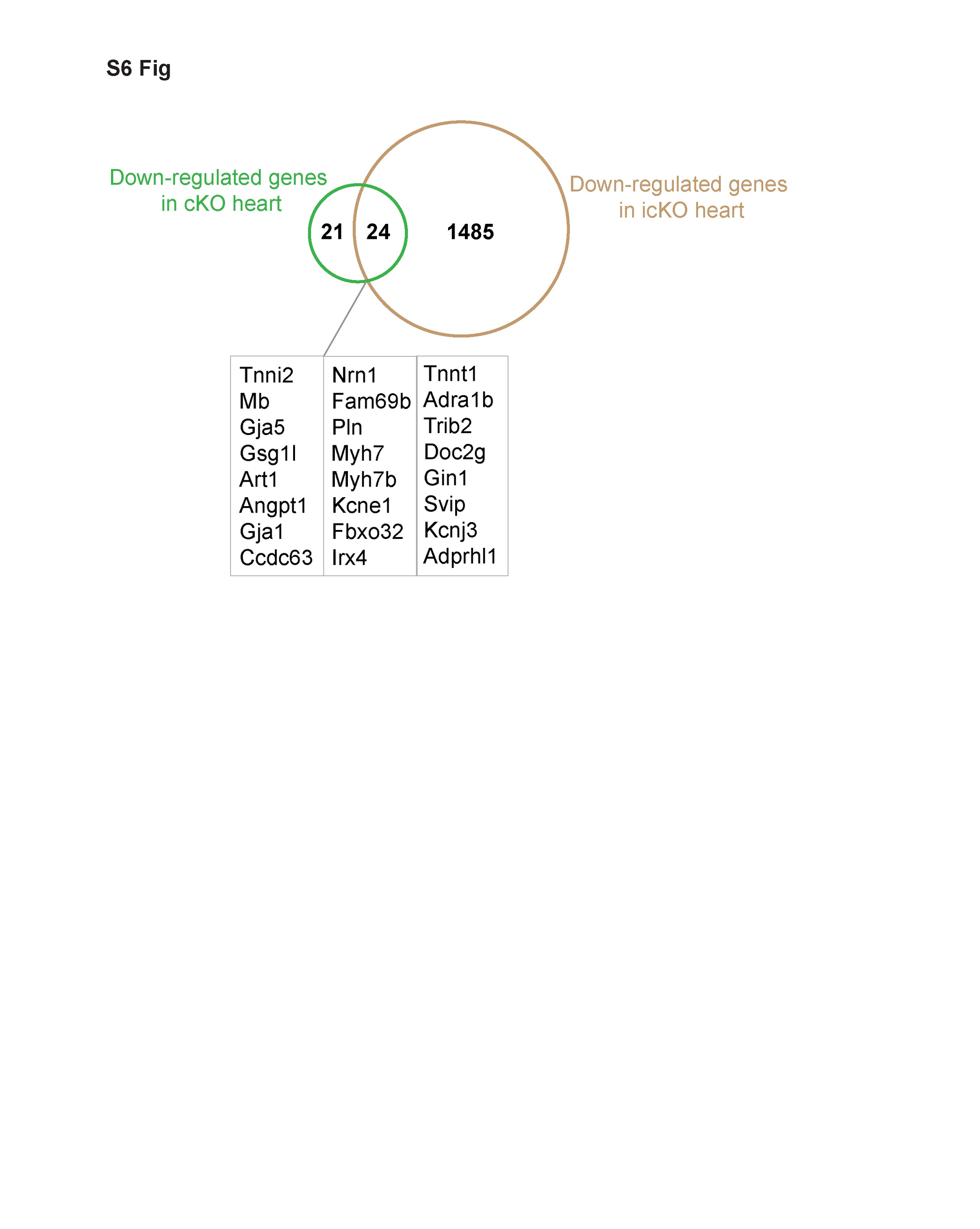

Supplement: S6 Fig — Venn diagram shows overlapping genes between down-regulated genes in Med30 cKO hearts (green) and icKO cardiomyocytes (brown). 24 overlapping genes were listed. (TIF) [file pgen.1009785.s006.tif]

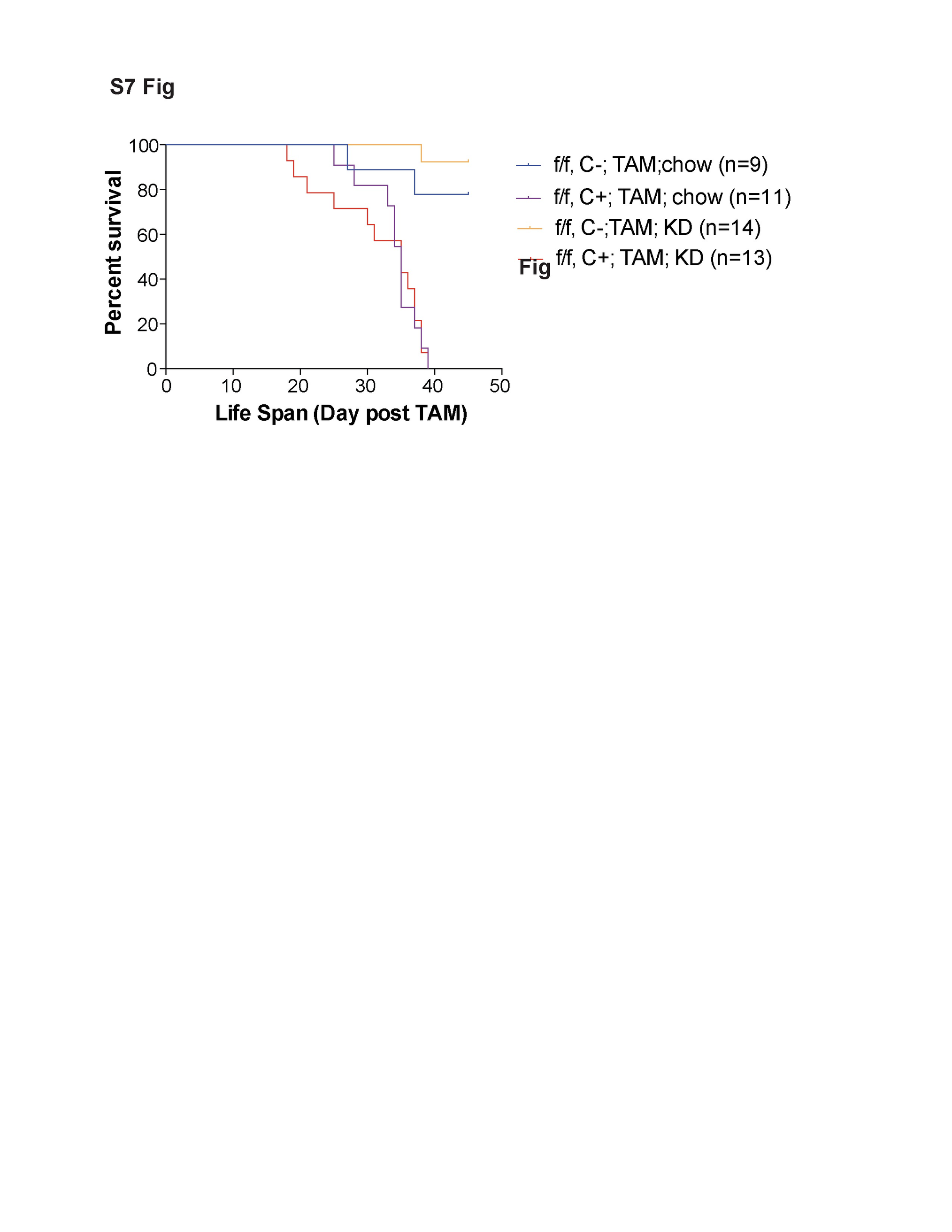

Supplement: S7 Fig — Kaplan-Meier survival curves of Med30 icKO and control fed with either normal chow or ketogenic diet (KD). n = 11–14 mice per group. (TIF) [file pgen.1009785.s007.tif]
