# Supplementary material for: Mediator complex proximal Tail subunit MED30 is critical for Mediator core stability and cardiomyocyte transcriptional network
Source: PLoS Genet. 2021 Sep 10;17(9):e1009785. doi: 10.1371/journal.pgen.1009785 (PMC8432849; doi:10.1371/journal.pgen.1009785)
Supplement: S1 Table — (PDF) [file pgen.1009785.s008.pdf]

S1 Table. Genotyping primers

Genomic DNA was extracted from mouse tails as previously described (55) , and polymerase chain reaction (PCR) was used to genotype the offspring using the following gene-specific primers (from 5′ to 3′):

**Primer for PCR:**		
**Gene**	**Forward**	**Reverse**
Med30 floxed	TTACCCCATACCTGGAAGACCT	GACACTTCTAGCGGTAATGAGC
Tnnt-Cre	GCACTCCAGCTTGGTTCCCGA	TACTCAAGAACTACGGGCTGC
αMHC-MerCreMer	GCCATAGGCTACGGTGTAAAAG	GTTGGTCAATAAGCCCATCATT
