# Supplementary material for: Mediator complex proximal Tail subunit MED30 is critical for Mediator core stability and cardiomyocyte transcriptional network
Source: PLoS Genet. 2021 Sep 10;17(9):e1009785. doi: 10.1371/journal.pgen.1009785 (PMC8432849; doi:10.1371/journal.pgen.1009785)
Supplement: S2 Table — (PDF) [file pgen.1009785.s009.pdf]

S2 Table. List of qRT-PCR primers

Primer for qRT-PCR:		
**Gene**	**Forward**	**Reverse**
Med30	CGTGCAGGACATCGTGTACC	CAGCTTTGTCCGGTCTT
Mycn	ACCATGCCGGGGATGATCT	AGCATCTCCGTAGCCCAATTC
Hey2	AAGCGCCCTTGTGAGGAAAC	GGTAGTTGTCGGTGAATTGGAC
Angpt1	CACATAGGGTGCAGCAACCA	CGTCGTGTTCTGGAAGAATGA
Gja1	ACAGCGGTTGAGTCAGCTTG	GAGAGATGGGGAAGGACTTGT
Gja5	GGTCCACAAGCACTCCACAG	CTGAATGGTATCGCACCGGAA
Tnnt2	CAGAGGAGGCCAACGTAGAAG	CAGAGGAGGCCAACGTAGAAG
Tnni2	CGGAGGGTGCGTATGTCTG	CAGGTCCCGTTCCTTCTCA
Kcnh2	GACACCATCATCCGCAAGTTT	CAGAAGCCGTCGTTGCAGTA
Kcnip2	GGATGAGTTTGAACTATCCACGG	GACAATTCCGCTGGGACATTC
KcnJ3	TGCGTGGCCAATGTCTATAA	CAGGATGGACTGGAAGAGGA
Cacna1c	CCTGCTGGTGGTTAGCGTG	TCTGCCTCCGTCTGTTTAGAA
Scn4a	AGTCCCTGGCAGCCATAGAA	CCCATAGATGAGTGGGAGGTT
Acadm	AGGGTTTAGTTTTGAGTTGACGG	CCCCGCTTTTGTCATATTCCG
Kcnk3	ACGGAGGCAAGGTGTTCTG	ACGACACGAAACCGATGAGC
Abcc9	CATAGCTCATCGGGTTCACAC	ATGTCTGCACGGACAAACGA
Acat1	CAGGAAGTAAGATGCCTGGAAC	TTCACCCCCTTGGATGACATT
Tnni3	TCTGCCAACTACCGAGCCTAT	CTCTTCTGCCTCTCGTTCCAT
Pln	AAAGTGCAATACCTCACTCGC	GGCATTTCAATAGTGGAGGCTC
Ryr2	ACGGCGACCATCCACAAAG	AAAGTCTGTTGCCAAATCCTTCT
Casq1	CTGGCACTGCTGTTTGTACTG	GGGGGCTCATGGTAGAGGAG
Tnnt1	CCTGTGGTGCCTCCTTTGATT	TGCGGTCTTTTAGTGCAATGAG
Mhy7	ACTGTCAACACTAAGAGGGTCA	TTGGATGATTTGATCTTCCAGGG
Atp2a2	TGGAACAACCCGGTAAAGAGT	CACCAGGGGCATAATGAGCAG
Mhy6	GCCCAGTACCTCCGAAAGTC	GCCTTAACATACTCCTCCTTGTC
Myom2	AAAAGACACAAGCACTTTGACCA	TGGGAGGATGACTGGGTGG
Rrad	GTCAGAGGAGGGCGTTTACAA	TCCACAGTGATAGAACGGTCA
Bckdhb	AGCTATTGCGGAAATCCAGTTT	ACAGTTGAAAAGATCACCTGAGC
Ucp3	CTGCACCGCCAGATGAGTTT	ATCATGGCTTGAAATCGGACC
Myoz2	GCATCAGCCATCACGAAGGAA	CCACGATTACTGAAATGGGACA
Cox10	AGAAGAGCTATACAGGGATTGCC	CTGTGTGACATACATGCGCTT
Ndufaf4	CACCGGAGTCAGTATCCAGAA	GGTTCAACTTTTACCGGCAAGG
Sdhb	AATTTGCCATTTACCGATGGGA	AGCATCCAACACCATAGGTCC
Pdk2	AGGGGCACCCAAGTACATC	TGCCGGAGGAAAGTGAATGAC
Uqcc1	AGTCCGAATGAAGCAGGAAG	GGGATTAACCCCCATGACTC
Slc25a13	CAGCCCAACCCGAAAACTGT	CTGGAACGCCACCATAAACAA
Lrrc10	CGACCGCTGCCAGAGTTATG	GTTGTCGCTGAGGTAGAGCTT
Tnnt2	CAGAGGAGGCCAACGTAGAAG	CTCCATCGGGGATCTTGGGT
ANF	GATAGATGAAGGCAGGAAGCCGC	AGGATTGGAGCCCAGAGTGGACTAGG
BNP	TGTTTCTGCTTTTCCTTTATCTGTC	CTCCGACTTTTCTCTTATCAGCTC
18s	GGAAGGGCACCACCAGGAGT	TGCAGCCCCGGACATCTAAG
