# Supplementary material for: Mediator complex proximal Tail subunit MED30 is critical for Mediator core stability and cardiomyocyte transcriptional network
Source: PLoS Genet. 2021 Sep 10;17(9):e1009785. doi: 10.1371/journal.pgen.1009785 (PMC8432849; doi:10.1371/journal.pgen.1009785)
Supplement: S3 Table — (PDF) [file pgen.1009785.s010.pdf]

S3 Table. List of Antibodies

Antibody	Source, Cat. No	Antibody	Source, Cat. No
donkey anti-rabbit A488	Thermo Fisher Scientific, A21206	goat anti-chicken A488	Thermo Fisher Scientific, A11039
donkey anti-rabbit A647	Thermo Fisher Scientific, A31573	donkey anti-rabbit A546	Thermo Fisher Scientific, A10040
donkey anti-goat A546	Thermo Fisher Scientific, A11056	donkey anti-mouse A546	Thermo Fisher Scientific, A10036
donkey anti-mouse A647	Thermo Fisher Scientific, A31571	Anti-Sarcomeric Alpha Actinin-RABBIT Monoclonal	Abcam, ab68167
RYR2	AbClonal, A0298	NKX2.5	Santa Cruz Biotech, sc-8697
MED30	Made from Dr. Thomas G. Boyer lab (35)	GAPDH	Santa Cruz, sc-32233
ATP2A2 / SERCA2	AbClonal, A1097	MED14 (CRSP2/DRIP150)	Bethyl, A301-044A
MED31	Proteintech, 16590-1-AP	MED8	Proteintech, 12182-1-AP
MED29	Abcam, ab177451	MED1	Bethyl
MED6	Proteintech, 15338-1-AP	MED20	Proteintech, 17598-1-AP
MED23/CRSP3	Bethyl, A300-425A-T	MED16	Bethyl, A303-668A-T
MED17	Life tech, PA530314	MED18	Bethyl, A300-777A
MED12	Bethyl, A300-774A	MED15	Bethyl, A301-278A-T
TRAP100/MED24	Bethyl, A301-472A	THRAP1/MED13	Bethyl, A301-278A-T
MED4	Made from Dr. Thomas G. Boyer lab (35)	KCNJ3	Alomone labs, APC-005
SCN4A	Alomone labs, ASC-020	CASQ1	Santa Cruz, sc-28274
CACNA1C	Alomone labs, ACC-003		
